# Ovarian Hormone-dependent and Spinal ERK Activation-regulated Nociceptive Hypersensitivity in Female Rats with Acid Injection-induced Chronic Widespread Muscle Pain

**DOI:** 10.1038/s41598-019-39472-z

**Published:** 2019-02-28

**Authors:** Ju-Hsin Chang, Shih-Ying Tsai, Yen-Jing Zeng, Yu-Cheng Liu, Chi-Yuan Li, Kuen-Bao Chen, Yeong-Ray Wen

**Affiliations:** 1 Graduate Institute of Clinical Medical Science, China Medical University, Taichung, Taiwan; 2 Department of Anesthesiology, China Medical University Hospital, Taichung, Taiwan; 3 Department of Anesthesiology, School of Medicine, China Medical University, Taichung, Taiwan; 4 Graduate Institute of Acupuncture Science, College of Chinese Medicine, China Medical University, Taichung, Taiwan; 5 Acupuncture Research Center, China Medical University, Taichung, Taiwan

## Abstract

Symptoms of chronic widespread muscle pain (CWP) meet most of the diagnostic criteria for fibromyalgia syndrome, which is prevalent in females. We used an acid injection-induced muscle pain (AIMP) model to mimic CWP. After female rats received an ovariectomy (OVX), acid saline solution was injected into the left gastrocnemius muscle. Time courses of changes in pain behaviours and p-ERK in the spinal cord were compared between groups. Intrathecal injections of oestradiol (E2) to the OVX group before two acid injections and E2 or progesterone (P4) injections in male rats were compared to evaluate hormone effects. We found that repeated acid injections produced mechanical hypersensitivity and enhanced p-ERK expression in the spinal dorsal horn. OVX rats exhibited significantly less tactile allodynia than did the rats in the other groups. The ERK inhibitor U0126 alleviated mechanical allodynia with lower p-ERK expression in the sham females but did not affect the OVX rats. Intrathecal E2 reversed the attenuated mechanical hypersensitivity in the OVX group, and E2 or P4 induced transient hyperalgesia in male rats. Accordingly, our results suggested that ovarian hormones contribute to AIMP through a spinal p-ERK-mediated pathway. These findings may partially explain the higher prevalence of fibromyalgia in females than males.

## Introduction

Musculoskeletal pain is a major issue that is often under-diagnosed and under-treated^1^. Ten percent of the general population experiences chronic generalized musculoskeletal pain, particularly fibromyalgia syndrome (FMS)^2,3^. FMS is characterized by widespread muscle pain with tenderness, morning stiffness, disturbed sleep and mood, and pronounced fatigue^2–4^. Females, compared with males exhibit a higher incidence of FMS^5^ as well as increased pain and poorer quality of life^6^.

Several hypotheses regarding pathophysiology of CWP have been proposed, such as central sensitization^7,8^; pain disinhibition^9,10^; peripheral sensitization^11^; increases in nociceptive substances^12^; up-regulation of an acid-sensing ion channel (ASIC3) in muscle^13^ and in dorsal root ganglia^14^; and elevated cytokines levels^15,16^. However, none of these pathways can adequately explain the sex difference in pain pathologies.

Sluka and colleagues have developed an animal model of chronic muscle hyperalgesia by repeated intramuscular injections of acidic saline^17^, which characteristically induces long-lasting and widespread mechanical hyperalgesia, central sensitization, and no inflammation or peripheral tissue damage^17–21^. Currently, this model is widely used to mimic CWP or FMS in humans.

The MAPK family includes ERK, p38, and c-Jun N-terminal kinase (JNK), which play critical roles in nociceptive processing^22^. Among them, ERK is activated after exposure to various noxious stimuli and is involved in different pathological states under conditions including spinal nerve ligation^23^; bladder distension^24^; and hind paw injection of complete Freund’s adjuvant^25^, formalin^26^, or carrageenan^27^; and visceral reflex activity^28,29^. More recently, ERK activity has also been identified in central amygdala^30^ and paraventricular thalamic nucleus anterior^31^ in AIMP. However, no studies have investigated the role of ERK activation in sex differences and the roles in CWP.

Increasing evidence indicates the crucial influences of female hormones on pain sensitivity^32,33^. Recently, a rigorous study has also demonstrated the effect of 17β-oestradiol (E2) on pronociception in an acetic acid-related pain model^34^. Female rats treated with physiological levels of oestrogen in the trigeminal ganglia exhibited altered gene expression, thus suggesting that oestrogen regulates genes potentially relevant to migraine^35^. Oestradiol, the most potent oestrogen, increased NMDA-evoked rat masseter muscle afferent discharge in a dose-dependent manner^36^ and potentiates nocifensive responses induced by capsaicin in ovariectomized rats^37^. In a chronic pain state, oestradiol increases allodynia via ERK activation in trigeminal nucleus caudalis neurons (TNC)^38,39^. Thus, we hypothesized that the ERK signalling pathway might regulate female hormone-related widespread muscle pain.

In the present study, we aimed to elucidate the role of ERK activation and female hormones in the AIMP model. Several approaches were conducted to compare nocifensive behaviours, time-dependent p-ERK expression between normal and ovariectomized rats, and the influence of p-ERK inhibition. In addition, we performed intrathecal supplementation of E2 in the OVX females and injection of E2 or progesterone (P4) in male rats to clarify the hormonal effects on AIMP between genders. Our results revealed that ovarian hormones contribute to AIMP through a spinal p-ERK-mediated pathway.

## Results

### Ovariectomy results in prolonged depletion of serum 17β-oestradiol

**Figure 1:**
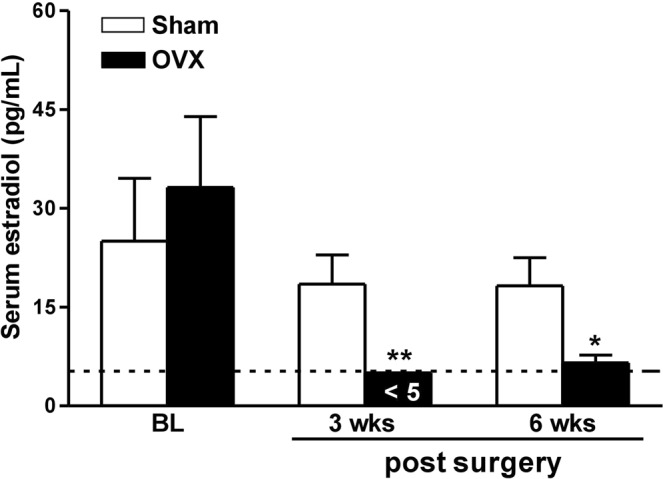
Serum oestradiol levels in sham operated and ovariectomized rats. The female rats received either bilateral ovariectomy (OVX) or sham operation (Sham). Serum oestradiol assays were performed before surgery (BL), prior to the 1st acid injection (3 weeks post-surgery), and 14 days after the 2nd acid injection (6 weeks post-surgery). The detection limit is 5 pg/mL for electrochemiluminescence immunoassay (dashed line). Values below the detection limit were arbitrarily reported as 5 pg/mL for the statistical analysis. *p < 0.05, **p < 0.01 vs. Sham by unpaired *t* test. Rat number = 6 for each group at individual time points.

No significant differences in serum concentrations of 17β-oestradiol were observed between the sham (Sham) and the ovariectomy groups (OVX) before surgery (baseline data 25.04 ± 9.52 pg/mL vs. 33.18 ± 10.74 pg/mL, p = 0.586, respectively, Fig. 1). Serum 17β-oestradiol was largely depleted at 3 weeks post-ovariectomy (on the day of the 1st acid injection), reaching undetectable levels (<5 pg/mL). OVX rats exhibited low 17β-oestradiol levels until 6 weeks post ovariectomy (on the 14th day after the 2nd acid injection, Fig. 1).

### Deprivation of ovarian hormones ameliorates repeated AI-induced widespread pain

**Figure 2:**
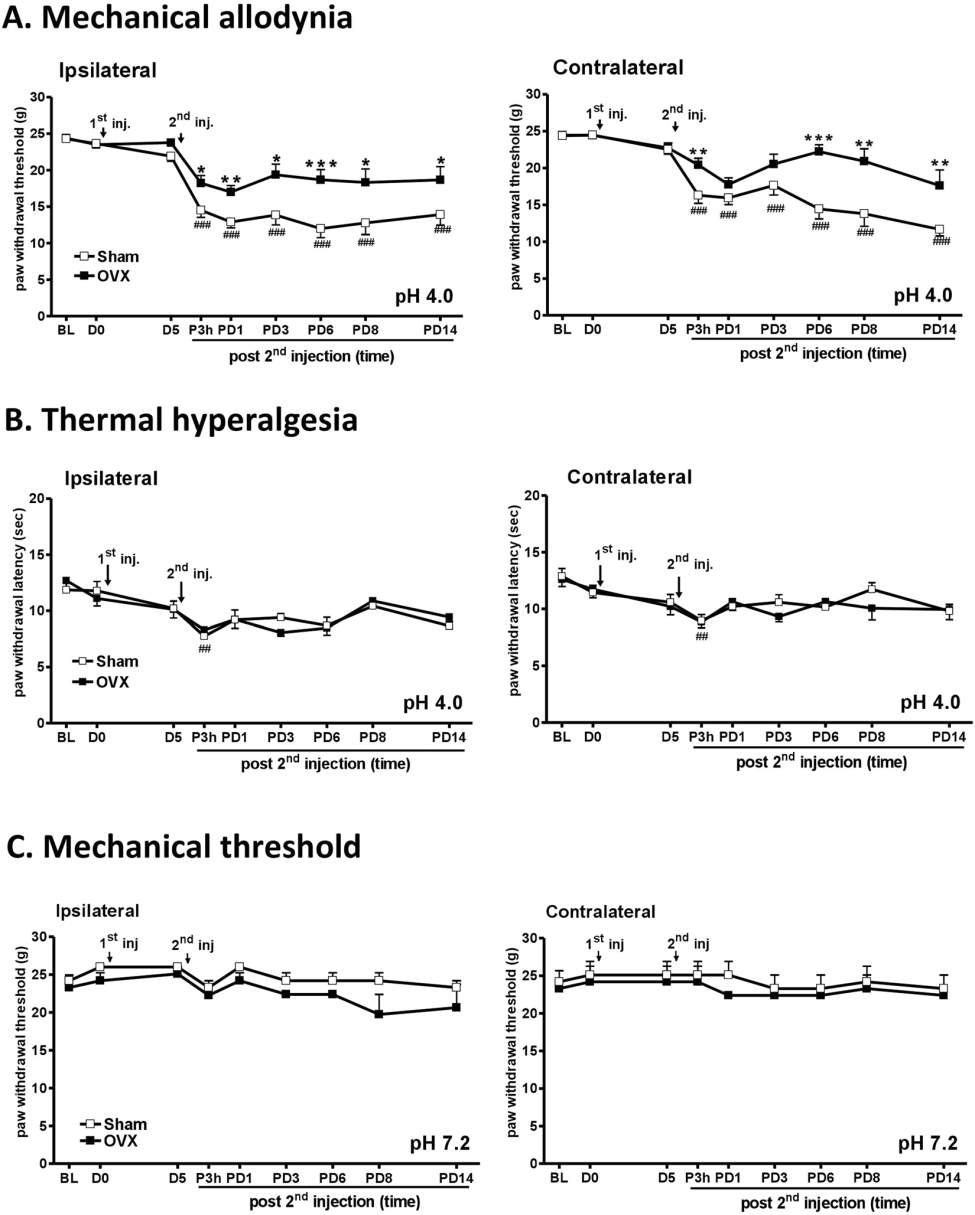
Ovariectomy mitigated mechanical allodynia but not heat hyperalgesia in a acid injection (AI)-induced widespread pain model. The female rats received either bilateral ovariectomy (OVX) or sham operation (Sham). Three weeks after surgery, rats received two AIs of 150 μl of HCl (pH = 4.0) or control saline (pH = 7.2) into the left gastrocnemius muscle on day 0 (D0) and day 5 (D5). Mechanical allodynia (**A**,**C**) and thermal hyperalgesia (**B**) of ipsilateral and contralateral hind paws are presented. Abbreviation, D: post 1st AI day; PD: post 2nd AI day; h: hour; arrows, indicating AIs. ^#^p < 0.05; ^##^p < 0.01; ^###^p < 0.001 vs. BL by two-way RM ANOVA followed *post hoc Tukey’s test*. *p < 0.05; **p < 0.01; ***p < 0.001 vs. Sham. Rat number = 8–12 for each group at individual time points.

Repeated pH 4.0 AI induced bilateral mechanical and heat hypersensitivities in the hind limbs in ovary-intact rats (Sham group) (Fig. 2A,B). Withdrawal thresholds of both hind paws in von Frey test were markedly lower starting 3 h after the 2nd AI (P3h) and were maintained at low levels over 14 days (PD14) (Fig. 2A). In addition, thermal hyperalgesia occurred at P3h in bilateral hind paws, but the decrease was mild as compared with mechanical thresholds (Fig. 2B). Of note, Fig. 2C shows that pH 7.2 saline injection did not significantly change withdrawal thresholds. These results revealed unilateral AI-induced bilateral or widespread nociceptive hypersensitivities.

In comparison, ovariectomy (the OVX group) mitigated the mechanical allodynia induced by repeated AI (Fig. 2A) but did not alter the basal von Frey thresholds (Fig. 2C). The differences between the OVX and Sham groups appeared at P3h to PD14 in bilateral hind paws (Fig. 2A). However, no group differences in heat hyperalgesia (Fig. 2B) were noted, thus suggesting that hormonal influence may be pain type-dependent.

### Repeated AI induces ERK activation in the spinal dorsal horn in a time-dependent manner

**Figure 3:**
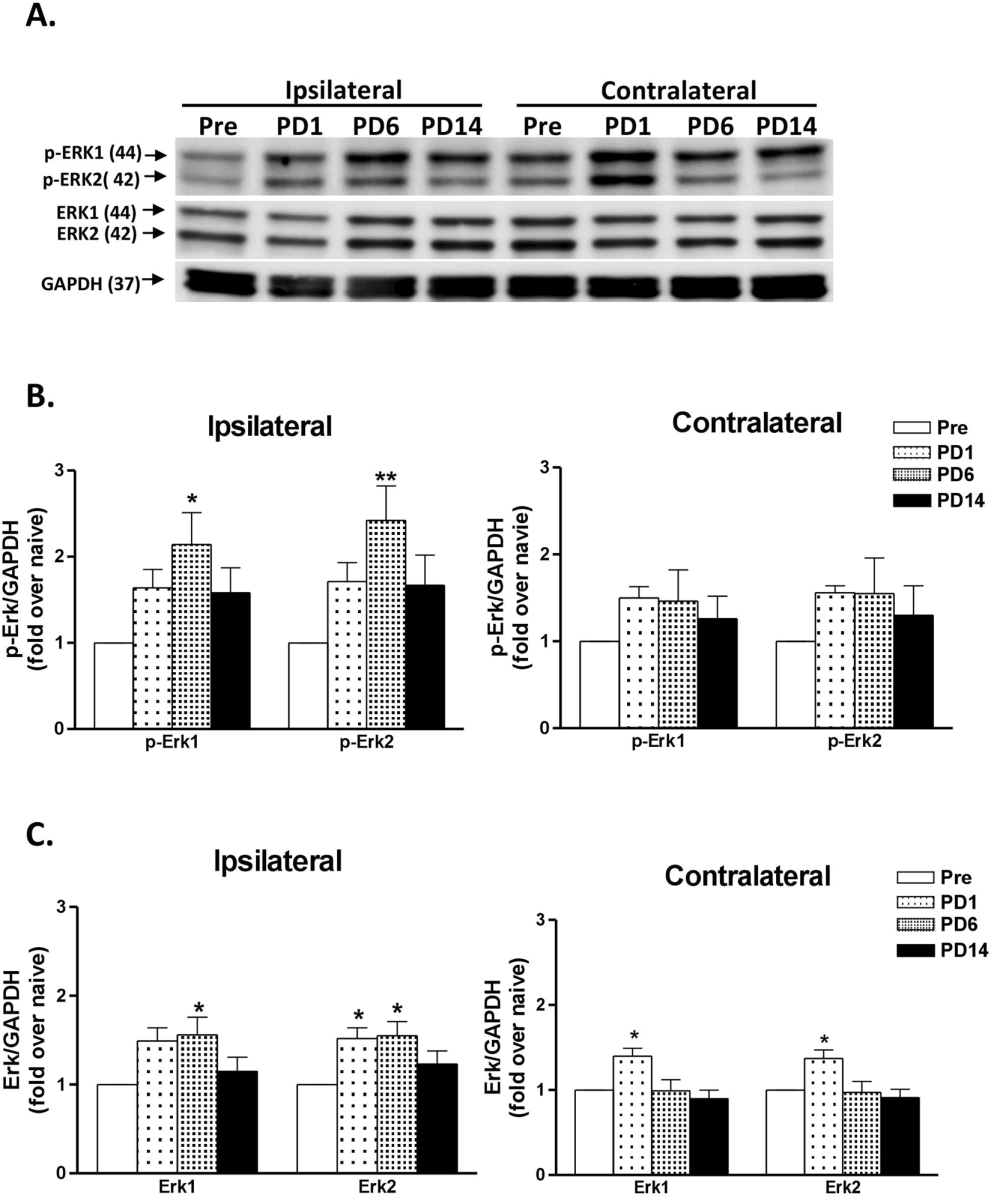
ERK1/2 and phosphorylated ERK1/2 in the lumbar dorsal spinal cord after intramuscular AI. (**A**) Western blot analysis reveals the time course of acid-induced p-ERK1/2 and ERK 1/2 expression in the spinal cords of ovary-intact rats. The western blot bands at the top showed the cropped target proteins. The full-length gel of the gel presented in this figure is shown in Supplementary Fig. S1 (S1-1 to S1-2). (**B**) The bar charts indicate the density levels of p-ERK1/2 bands after normalization to GAPDH. (**C**) The bar charts indicate the density levels of ERK1/2 bands after normalization to GAPDH. Data are presented as fold-change compared with pre-injection rats (Pre). PD, day post 2nd injection day. *p < 0.05; **p < 0.01, vs. Pre by one-way ANOVA followed *post hoc* Tukey’s test. Rat number = 5 at individual time points.

ERK1/2 (p-ERK) activation and ERK1/2 were observed in the lumbar dorsal cord after AI. Western blot analysis revealed time-dependent changes in acid-induced ERK1/2 and p-ERK1/2 in the spinal cord in the rats. (Fig. 3A) Phosphorylated ERK1/2 increased on day 1 after the 2nd injection, peaked at day 6, and declined at day 14 in the ipsilateral side (Fig. 3B). Significant differences were noted in the PD6. However, at the contralateral side, an increasing trend was observed, but the values did not significantly differ. ERK1/2 increased in the ipsilateral dorsal horn on day 1 and 6 after the 2nd injection, and only day 1 in the contralateral dorsal horn after the 2nd injection (Fig. 3C).

### Deprivation of ovarian hormones decreases AI-induced ERK activation in the spinal horn

**Figure 4:**
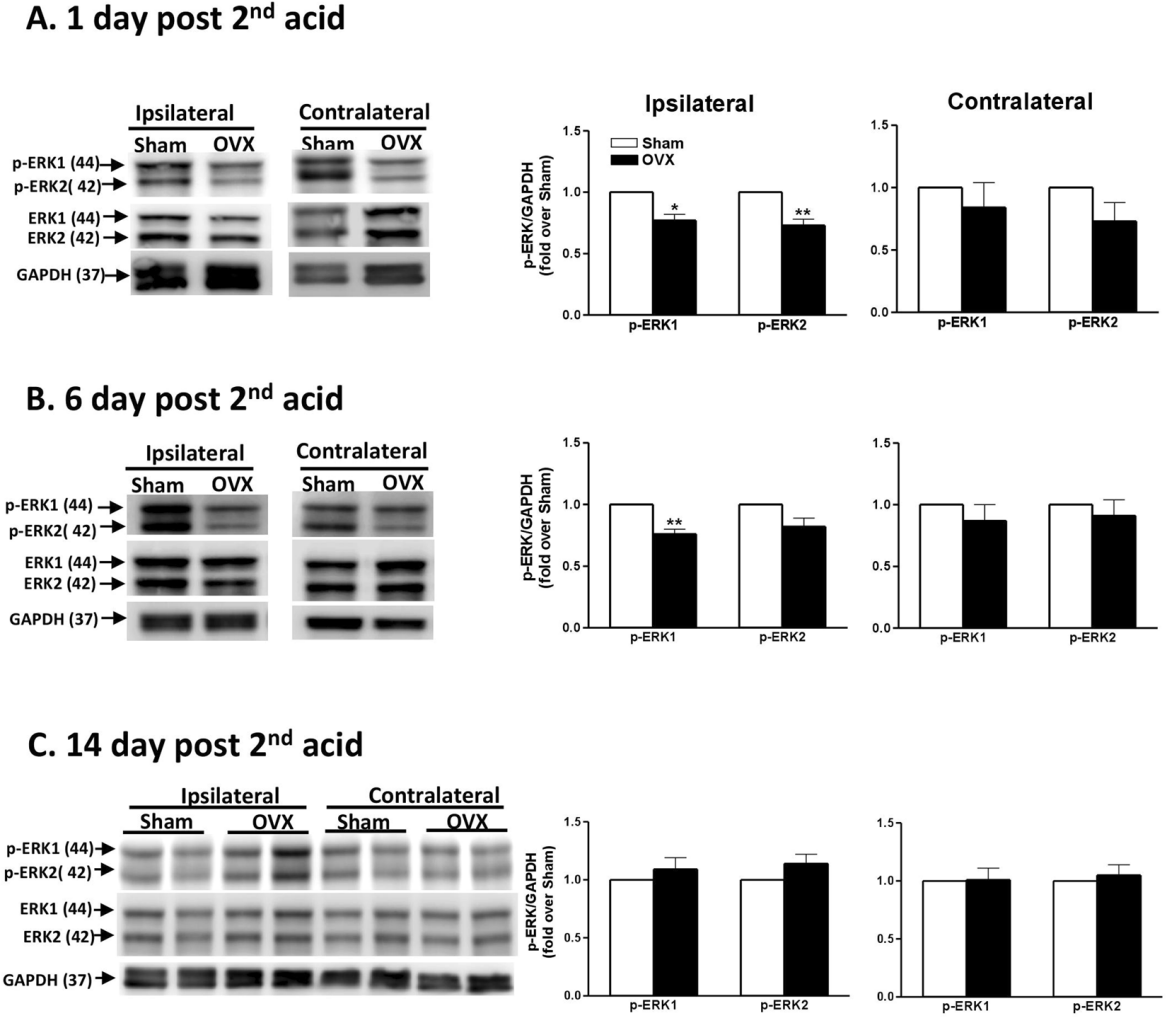
Influence of ovariectomy on AI-induced ERK activation. Western blots (left) and quantification (right) of p-ERK1/2 and ERK1/2 in the dorsal cords of the sham and OVX rats on days 1, 6, and 14 after the 2nd AI are presented. In the right panels, the densities of p-ERK1/2 bands are normalized to GAPDH and presented as fold-change compared with sham rats. The full-length gel of gels presented in this figure is shown in Supplementary Fig. S2 (S2-1 to S2-6) and quantification of ERK1/2 is in Supplementary Fig. S3. *p < 0.05; **p < 0.01 vs. sham by unpaired *t* test. Rat number = 4–6 for each group at individual time points.

Western blotting and immunostaining were used to evaluate the effects of ovarian hormones on AI-induced ERK activation. In Fig. 4, OVX rats, compared with the Sham rats, exhibited lower p-ERK1/2 on day 1 (PD1) and lower p-ERK1 on day 6 (PD6) at the AI-side dorsal horn (Fig. 4), but no differences were observed on day 14 (PD14). Of note, no differences in p-ERK1/2 at the contralateral dorsal horn were observed at any time point. There was no difference of ERK1/2 expression between groups at any time points (Fig. S3).

**Figure 5:**
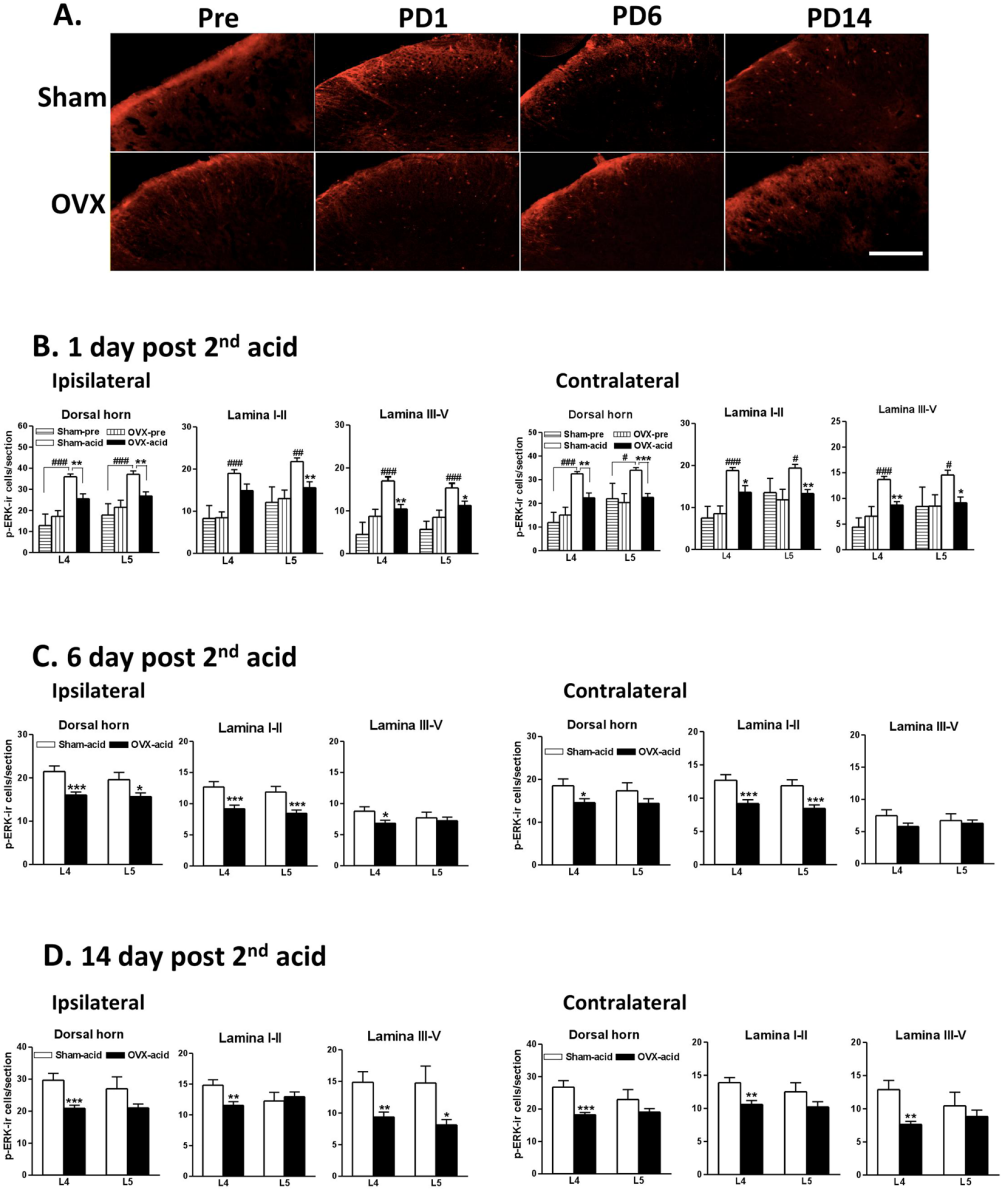
Effect of ovariectomy on AI-induced ERK activation in the spinal dorsal horn. (**A**) Immunofluorescence reveals the p-ERK distribution in the L4/5 ipsilateral spinal cords of the Sham and OVX groups before the 1st AI (Pre) and on days 1, 6, and 14 post 2nd AIs (PD). Upper panels: Sham; lower panels: OVX. Scale bar: 100 μm. (**B**–**D**) Comparisons of p-ERK-ir cell numbers among groups. Data were analysed on the basis of laminae, spinal segment, injection side, and different time points. One-way ANOVA with *post hoc* Tukey’s test for (**B**) and unpaired *t* test for (**C**,**D**), ^#^p < 0.05, ^##^p < 0.01, ^###^p < 0.001 *vs*. sham-pre; *p < 0.05, **p < 0.01, ***p < 0.001 between groups in (**B**) or *vs*. sham-acid in (**C**,**D**). Rat number = 3–5 for each group at individual time points. Spinal section number = 30–35 for each group from 3–5 rats at individual time points.

To understand the distribution of ERK activation, AI-induced p-ERK-immunoreactivity at the L4 and L5 spinal segments were assessed. In Fig. 5A, immunofluorescence indicated that p-ERK-ir cells appeared on day 1 after AI and were predominantly located in the outer third of the superficial lamina (laminae I and II). Then, the cells were equally distributed at the superficial and deep (lamina III-V) layers on day 14 (Fig. 5A). Our findings were consistent with findings from numerous studies suggesting that p-ERK appears in the superficial laminae at very early stages after noxious stimulations and persists in the spinal dorsal horns^23^.

The immunofluorescence results revealed clear p-ERK changes. OVX rats, compared with the sham group, exhibited significantly lower p-ERK-ir cells in bilateral L4-L5 on days 1, 6, and 14 (Fig. 5B–D). OVX rats exhibited strong inhibition in both side dorsal horns on PD1 (Fig. 5B) and moderate inhibition at the superficial laminae on PD6 and at the deep laminae (III-V) on PD14 (Figs 5C,D, respectively). Notably, the distribution patterns of p-ERK were similar between the sham and OVX groups, on the basis of segments, sides, lamina, or time points. No significant difference in p-ERK was observed between groups before AI (Sham-pre vs. OVX-pre, Fig. 5B).

### AI-induced p-ERK-ir cells are spinal neurons or astrocytes

**Figure 6:**
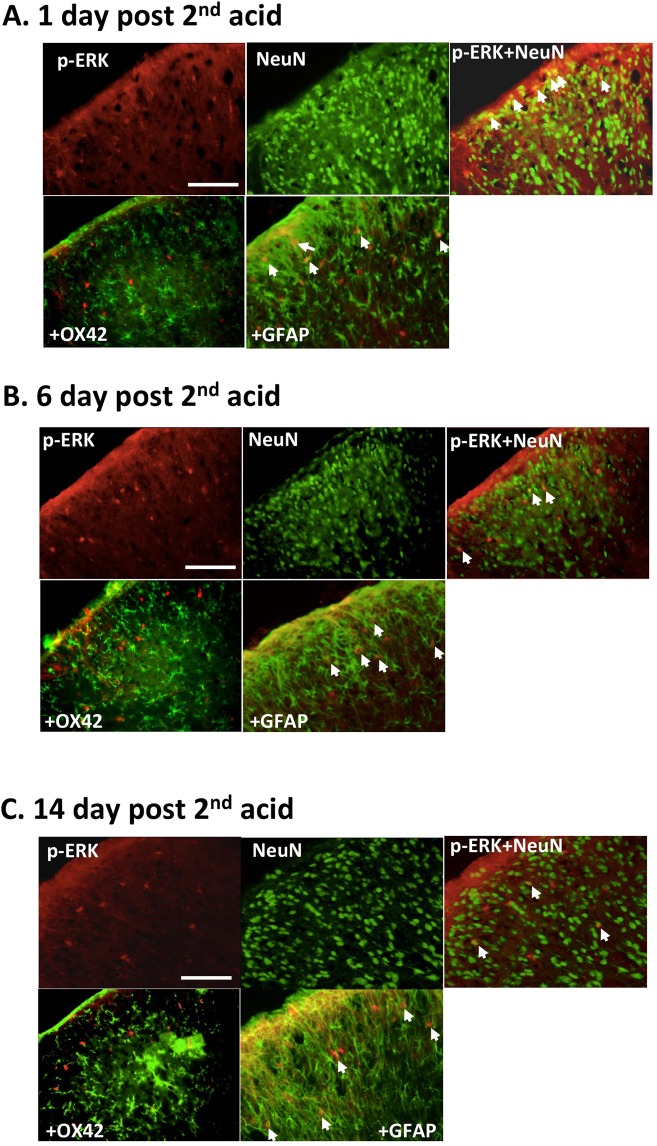
Temporal and cell-type profile of p-ERK expression after repeated AIs. Immunofluorescence is depicted in red for p-ERK; green for NeuN (neuron), OX-42 (microglia), or GFAP (astrocyte) staining; and orange-yellow for colocalization. Double labelling through days 1 (**A**), 6 (**B**), and 14 (**C**) after the 2nd acid injection. p-ERK is exclusively colocalized with NeuN and GFAP not OX42. White arrows: colocalization; scale bar: 50 µm.

On the basis of the time sequence, double staining demonstrated that p-ERK colocalized with NeuN (neurons) in the superficial laminae (I-II) and with NeuN or GFAP (astrocyte) in dorsal laminae (I-IV) throughout days 1, 6, and 14 post-AI (Fig. 6A–C). We also observed that p-ERK was first expressed within neurons on PD 1 and both astrocytes and neurons on PD14 (Fig. 6A–C). Co-expression of p-ERK and OX42 (microglia) was not noted at any time point.

### Inhibition of ERK activation reverses AI-induced mechanical allodynia

**Figure 7:**
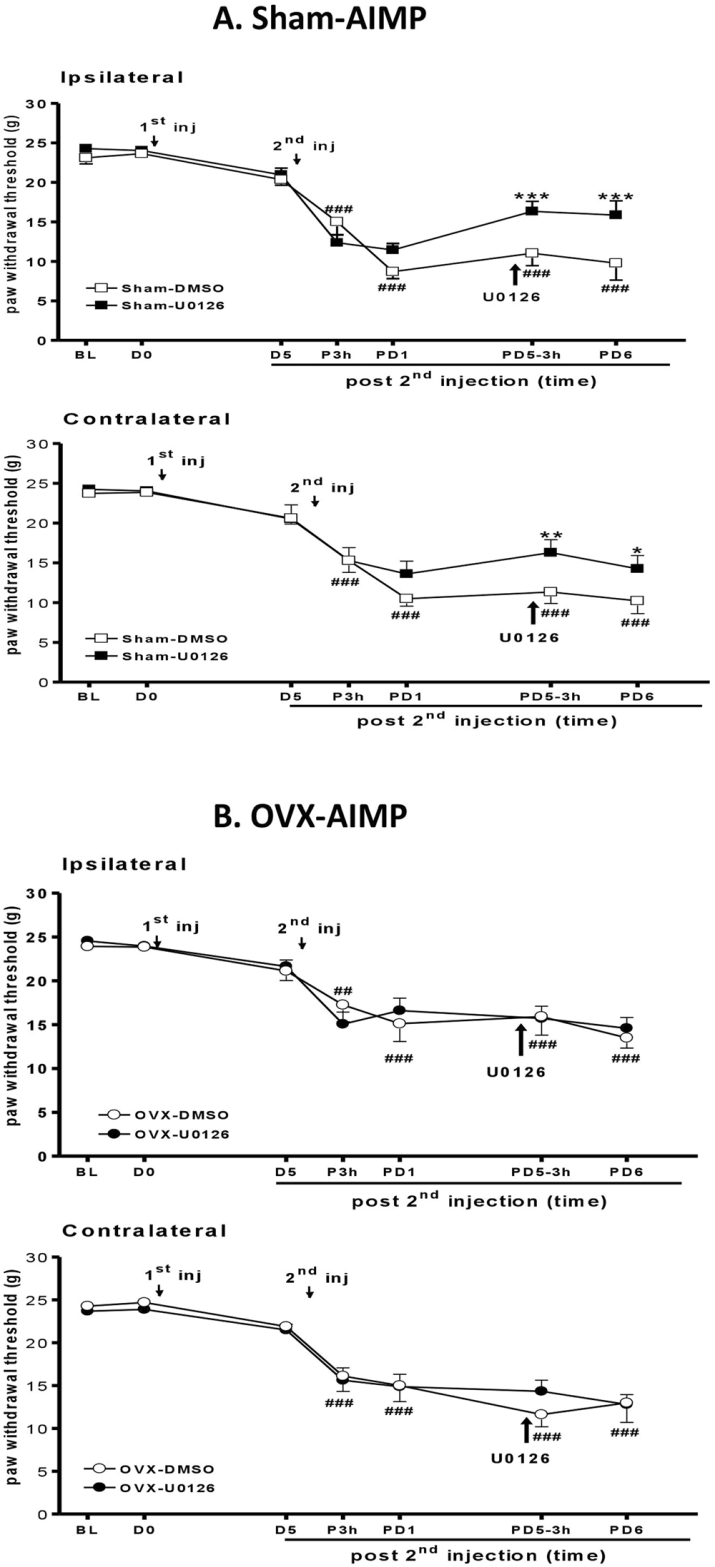
U0126 reversed acid-induced mechanical allodynia in the sham but not OVX rats. (**A**) Intrathecal (i.t.) injection of the ERK inhibitor U0126 (10 μg in 20 μL 5% DMSO) in the ovary-intact (Sham) rats on day 5 after the 2nd AI (PD5) attenuated mechanical allodynia at 3 h and the next day (PD6). This effect was not detected in vehicle-treated rats (Sham-DMSO). (**B**) No difference was observed in OVX rats with i.t. U0126 or vehicle injection. BL, before 1st injection; PD1, day 1 post 2nd AI; PD5-3 h, 3 h post U0126/or vehicle injection; PD6, day 6 post 2nd AI. ^##^p < 0.01; ^###^p < 0.001 *vs*. BL by two-way RM ANOVA with *post hoc* Tukey’s test; *p < 0.05; **p < 0.01; ***p < 0.001 *vs*. DMSO. Rat number = 5–8 for each group at individual time points.

We also injected the ERK inhibitor in control and OVX rats to compare the contribution of p-ERK. We found that the ovary-intact rats treated with i.t. U0126 at a dose of 10 μg on PD5 exhibited less AI-induced mechanical allodynia in the ipsilateral paw at 3 h after injection (Fig. 7A). In contrast, U0126-treated OVX rats had no evident allodynic suppression (Fig. 7B). Because the heat threshold did not clearly change in OVX rats, we did not compare the effect of U0126 on thermal thresholds. All results suggested that p-ERK may play a pivotal role in mediating ovarian hormone-dependent, AI-induced mechanical hypersensitivity.

**Figure 8:**
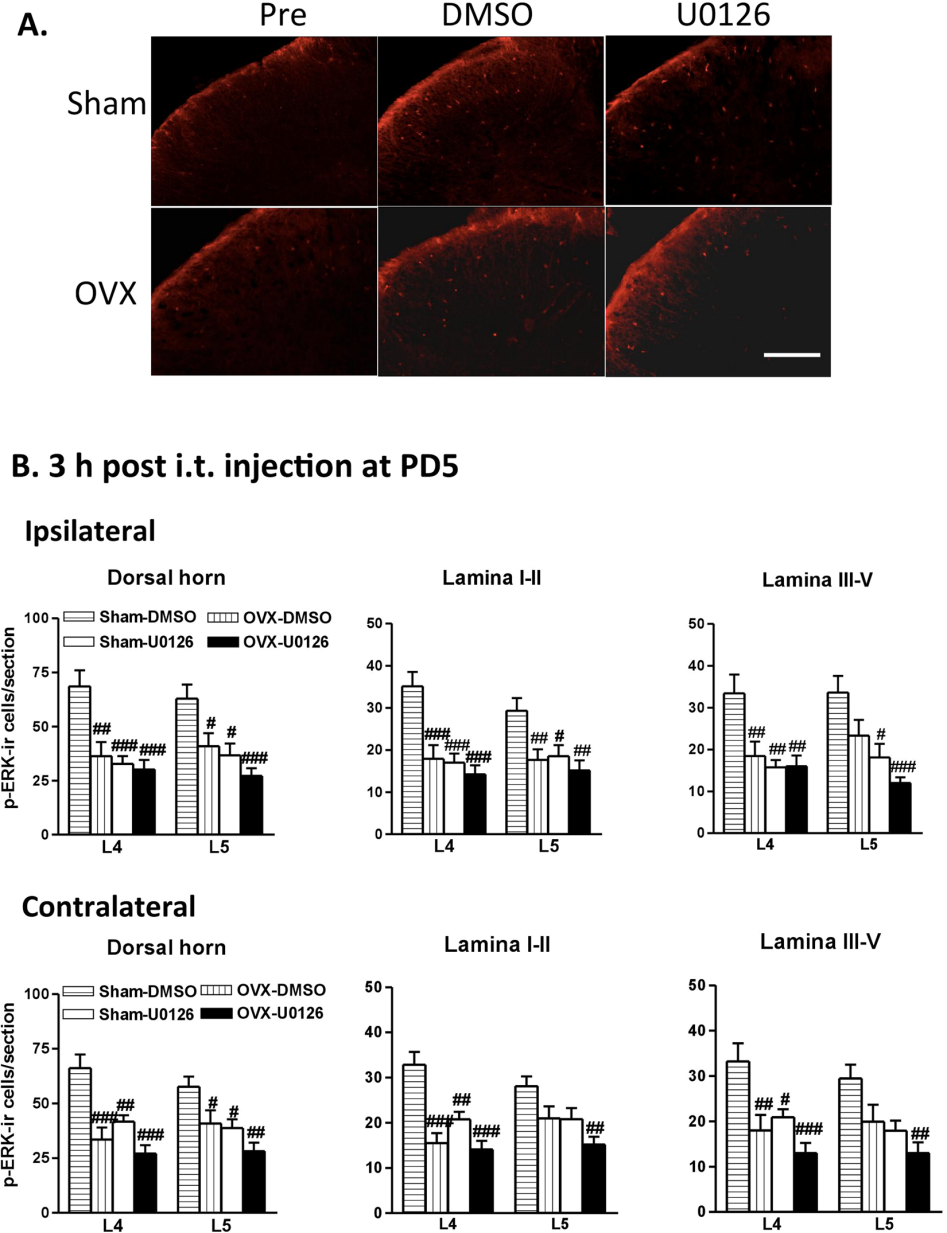
Immunofluorescence analysis indicates the effect of ERK inhibitor on acid injection-induced ERK activation in the spinal dorsal horn. U0126 or vehicle (doses as Fig. 7) was injected 5 days after the 2nd AI. (**A**) Immunofluorescence reveals the p-ERK distribution in the L4/5 ipsilateral spinal cords before the 1st AI (Pre) and 3 h post U0126 or DMSO injection. Upper panels: Sham; lower panels: OVX. Scale bar: 100 μm. (**B**) Comparisons of p-ERK-ir cell numbers among the sham and OVX groups with DMSO or U0126. Data were analysed on the basis of laminae, spinal segment, and AI side. One-way ANOVA with *post hoc* Tukey’s test, ^#^p < 0.05, ^##^p < 0.01, ^###^p < 0.001 vs. Sham-DMSO. Rat number = 3–6 for each group.

We also examined p-ERK expression after i.t. U0126. Fluorescence staining revealed that i.t. U0126 significantly decreased p-ERK-immunoreactivity in the Sham groups (Sham-DMSO vs. Sham-U0126, Fig. 8B) but not in the OVX groups (OVX-DMSO vs. OVX-U0126, Fig. 8B). U0126 exhibited similar effects on p-ERK in the contralateral dorsal horns (Fig. 8B).

### Male rats display higher mechanical thresholds and lower p-ERK1/2 expression in AIMP

**Figure 9:**
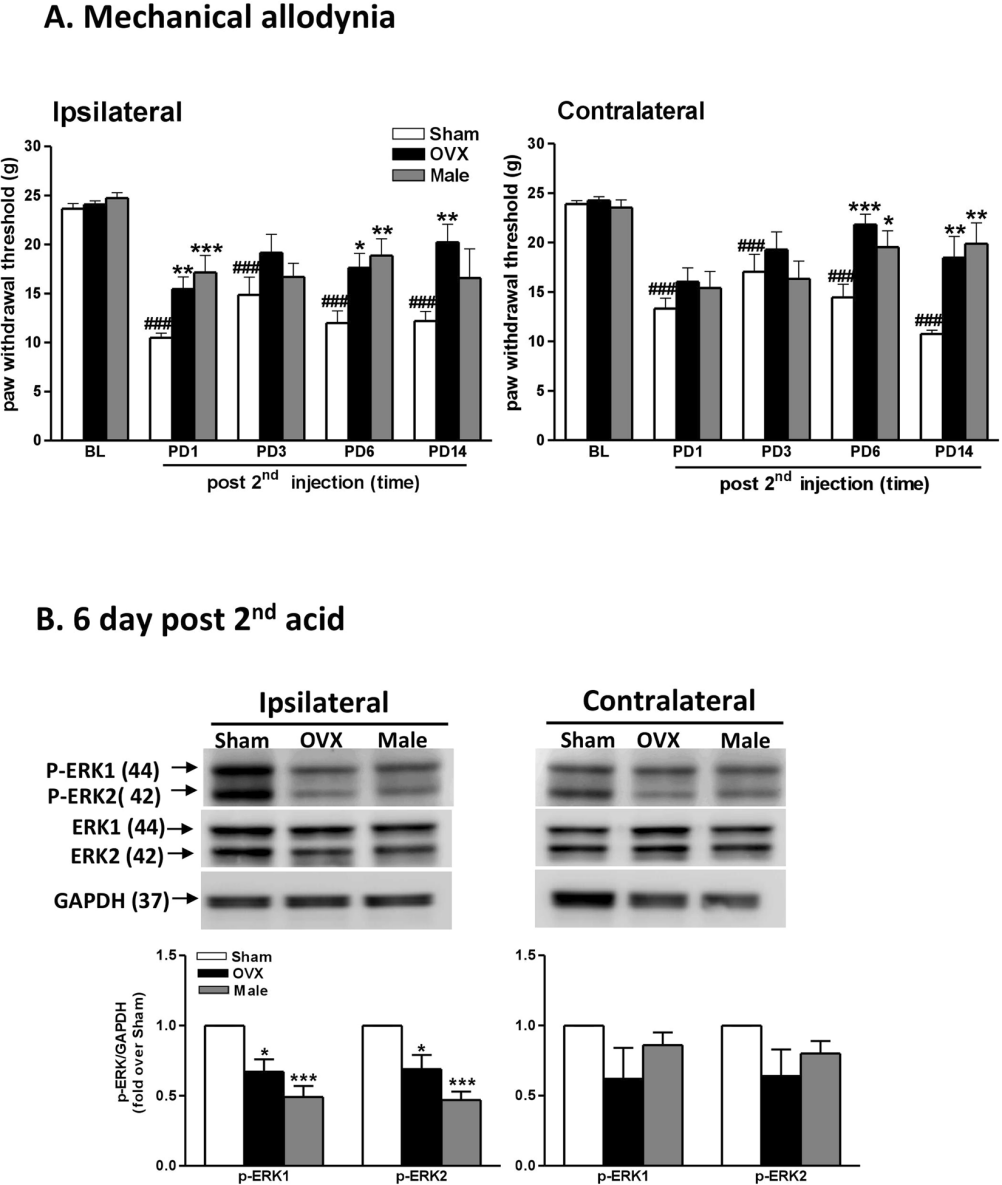
Male rats displayed a higher allodynic threshold and lower p-ERK1/2 expression after AIs than did female rats. (**A**) Behavioural tests were performed before the 1st injection as baseline (BL) and different days after the 2nd injection (PD) among the ovariectomized females (OVX), sham-operated females (Sham), and sham-operated males (Male). Mechanical withdrawal thresholds of ipsilateral and contralateral hind paws are presented. ^###^p < 0.001 *vs*. BL in Sham group; *p < 0.05; **p < 0.01; ***p < 0.001 *vs*. Sham by one-way ANOVA followed by *post hoc* Tukey’s test. Rat number = 8–12 for each group at individual time points. (**B**) Western blot analysis revealed acid-induced p-ERK1/2 and ERK 1/2 expression among three groups on day 6 post-2nd AI. The western blot bands at the top showed the cropped target proteins. Lower panels show the density levels of p-ERK1/2 bands after normalization to GAPDH. The bands in groups were run together in the same gel. GAPDH was used as the internal control in the same gel and at the same time. Data are presented as fold-change compared with Sham. *p < 0.05; ***p < 0.01 *vs*. Sham by one-way ANOVA followed by *post hoc* Tukey’s test. Rat number = 5 for each group. The full-length gels of the presented gels in this figure are shown in Supplementary Fig. S4 (S4-1 to S4-2) and the quantification of ERK1/2 are shown in Supplementary Fig. S5.

To evaluate the influence of sex in AIMP and ERK activation, we compared nociceptive responses and spinal p-ERK expression in female rats with (OVX) or without (Sham) ovariectomy and male rats after AI. Male rats exhibited levels of withdrawal responses similar to those of the OVX rats, whereas higher withdrawal thresholds than those of the female sham rats were observed in both hind paws (Fig. 9A). We also analysed AI-induced p-ERK and found that male and OVX rats exhibited similar p-ERK expression on day 6 post-AI (PD6), and these levels were significantly lower than those in the female sham rats in the ipsilateral area. No contralateral side differences in p-ERK expression were observed (Fig. 9B). There was no difference of ERK1/2 expression between three groups. (Fig. S5 in the Supplementary File).

### Mechanical allodynia induced by intrathecal injections of E2 and P4 in male rats

**Figure 10:**
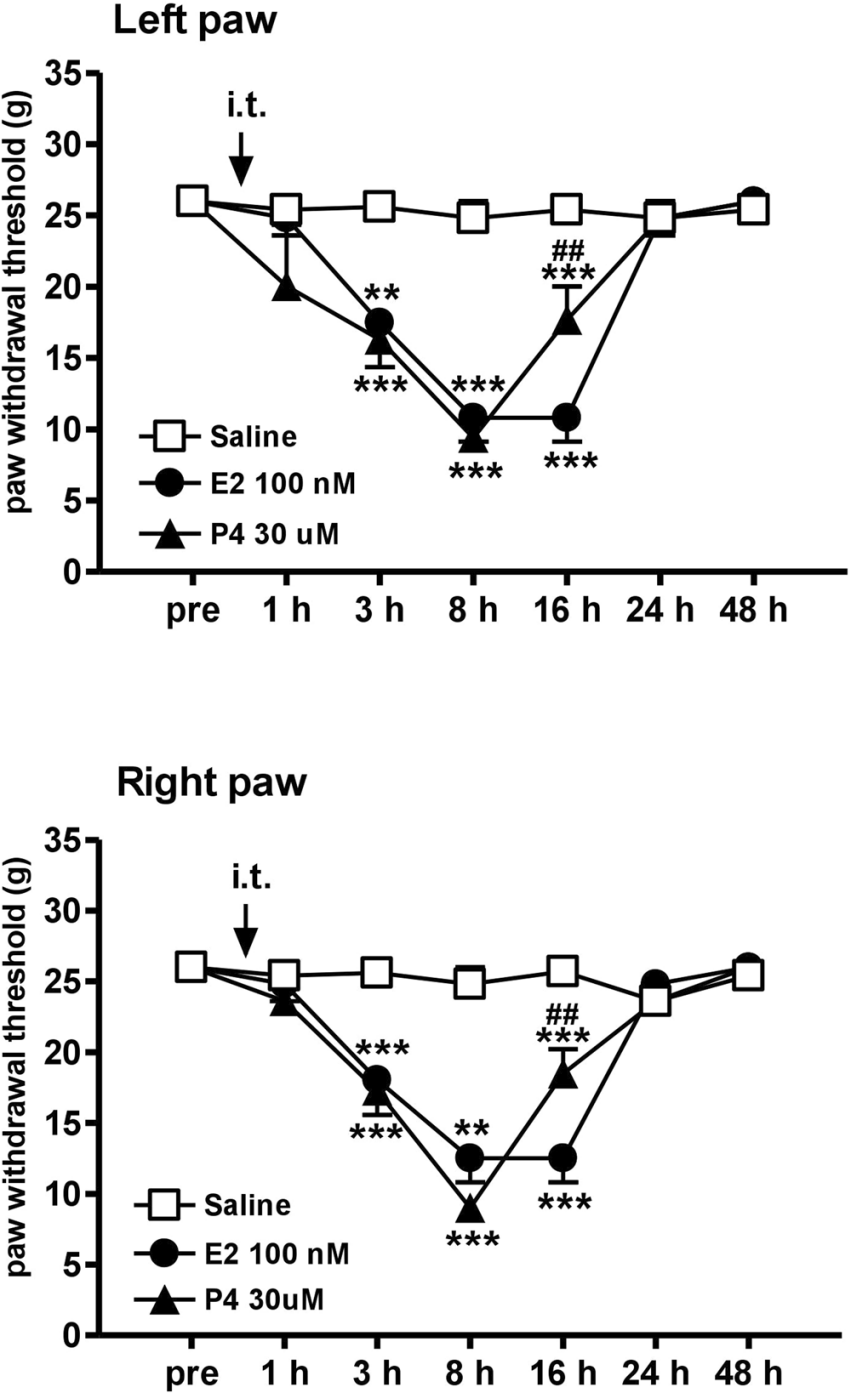
Intrathecal injections of E2 and P4 in male rats produced transient mechanical allodynia. I.t. injection of saline (10 μL) exhibited no effect on the tactile threshold but i.t. E2 (100 nM, 10 μL) or P4 (30 μM, 10 μL) induced mechanical allodynia in male rats. **p < 0.01; ***p < 0.001 *vs*. saline; ^##^p < 0.01; ^##^p < 0.01 for P4 vs. E2 by one-way ANOVA followed by *post hoc* Tukey’s test. Rat number = 5 for each group.

To determine whether ovarian hormones affect spinal nociceptive sensitization, we administered E2 (100 nM, 10 μL) and P4 (30 μM, 10 μL) intrathecally in the male rats. Bilateral hind paw withdrawal thresholds were markedly decreased after E2 or P4 injection at 3 h, reached the lowest level at 8 h, and then returned to baseline by 24 h (Fig. 10). In comparison, saline injection did not alter withdrawal thresholds (Fig. 10). These findings demonstrated that male rats treated with intrathecal ovarian hormones developed acute and short mechanical hypersensitivity for at least 24 h.

### Replenishing ovarian hormone via intrathecal E2 recovers hypersensitivity in OVX rats

**Figure 11:**
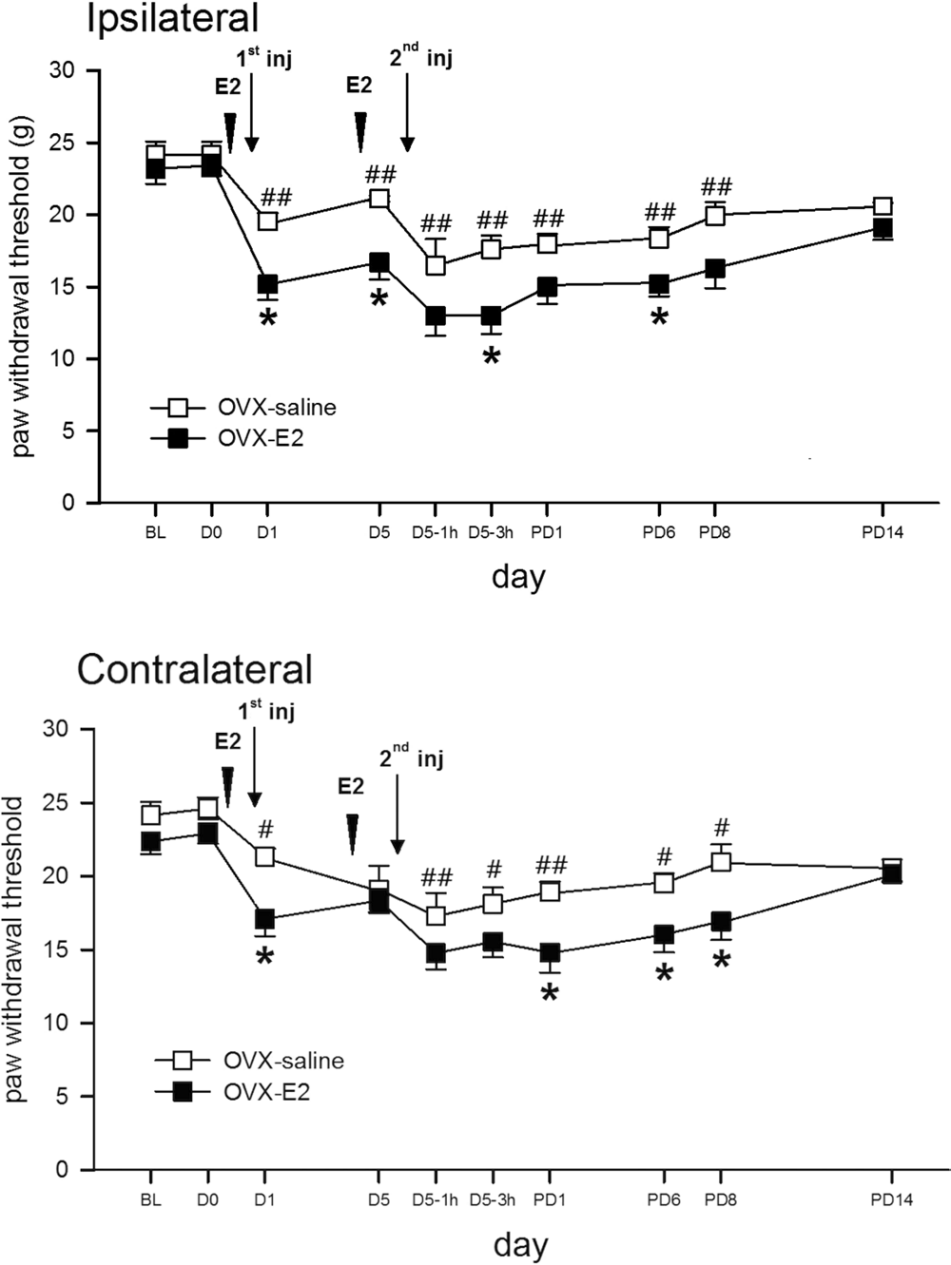
Intrathecal injections of E2 (100 nM, 10 μL), compared with saline (10 μL) injection in OVX female rats enhanced mechanical allodynia after AIs. Bilateral hind paws exhibited a similar trend of withdrawal thresholds. BL, Baseline data; D0, the day for the 1st AI; D5, 5 days after 1st AI; D5-1h, 1 h post 2nd acid injection on D5; D5-3 h, 3 h post 2nd AI; PD, post 2nd AI day; h, hours; arrows, acid injection; arrow heads, E2 or saline injection; *p < 0.05 vs. E2 by unpaired *t* test; ^#^p < 0.05; ^##^p < 0.01 *vs*. BL by two-way RM ANOVA followed by *post hoc* Tukey’s test. Rat number = 8 for each group at individual time points.

We treated the OVX females with i.t. E2 to assess whether replenishment of ovarian hormones would restore AI-induced pain. OVX females exhibited similar pain behaviours as that of the sham group after two i.t. E2 supplements (Fig. 11). Each E2 injection hypersensitized AI-induced withdrawal responses in the OVX rats back to levels analogous to those of normal female rats. The recovered sensitization persisted for at least 6 days and was present in bilateral hind paws.

## Discussion

In the present study, we described ovary hormone-dependent mechanical hypersensitivity in bilateral hind paws after repeated acid injections (AIs) in the left gastrocnemius muscle. Ovariectomy decreased injection-induced bilateral allodynia and ERK activation in the spinal dorsal horn. Intrathecal injection of a p-ERK inhibitor attenuated AI-induced widespread muscle pain in the ovary-intact rats but had no effect on OVX rats. Male rats exhibited similar levels of spinal p-ERK to those in OVX after repeated AI, and exhibited temporary mechanical allodynia when they received intrathecal injection of one of two main ovarian hormones, E2 and P4. Intrathecal injection of E2 also enhanced AI-induced withdrawal responses in the OVX rats, but had no effect to normal female rats. Together, these findings indicated the roles of ovarian hormones and spinal ERK activation in nociceptive plasticity of repeated AI-induced muscle pain.

Sluka’s AIMP model can mimic fibromyalgia with central nociceptive sensitization and presentations, such as chronic, widespread, non-inflammatory pain and signs, such as fatigue^40,41^. These features are comorbid with anxio-depressive behaviours^42^. We demonstrated that after repeated AI with pH 4.0 solution but not pH 7.2 solution, the bilateral hind paws exhibited persistent hypersensitivity to mechanical stimulation for at least 14 days. These results are comparable to findings from Sluka’s report and others^17,43,44^. Our findings justify the application of this model to investigate the influence of sex on widespread pain.

First, we demonstrated that ovariectomy significantly attenuated mechanical allodynic responses in female rats, whereas OVX rats preserved mild AI-induced mechanical hypersensitivity, thus suggesting that ovarian hormones amplify the hyperalgesic state in AI-induced pain. Moreover, we found that E2 supplementation by intrathecal injection restored mechanical hypersensitivity in OVX rats. However, ovariectomy only partially reverse AIMP, by half. We also found that both ovarian hormones, E2 and P4, are involved, because intrathecal injection of either hormone in male rats induced mechanical hypersensitivity. Of particular note, ovariectomy per se did not alter the basal mechanical and thermal thresholds, thus indicating that the normal presence of female hormones may not determine sex differences in nociceptive thresholds. A similar concept has been postulated previously^41^.

Ovariectomy deprives normal sources of ovarian hormone secretion in reproductive females. We found that serum 17β-oestradiol was almost completely deprived 3 weeks after surgery and maintained at low levels for 6 weeks. The mild rebound of oestradiol levels from 3 to 6 weeks is not surprising, because ovariectomy causes increases in body weight and enrichment of fat^45^. Adipose tissue is another main source of peripheral oestrogens, given that high expression of the enzyme aromatase can convert androgens to oestrogens^46^. We therefore hypothesize that peripheral oestradiol secretion may have compensated for the deprivation and may have diminished the difference between OVX and control animals in the long term.

Clinically, female adults tend to have more pain-related diseases and are prone to higher grades of pain after injuries than do male adults. Numerous types of chronic pain are more prevalent in women, such as chronic tension headache^47^, migraine^48^, facial pain^49^, temporomandibular joint (TMJ) pain^50^, musculoskeletal pain^51,52^, and fibromyalgia^53^. Numerous animal studies have also revealed that female rodents, compared with male rodents, exhibit higher susceptibility to noxious stimuli, such as hind paw injection of formalin^54^, CFA^55^, or tail injection of capsaicin^56^. However, such a sex difference is not conclusive, because male rodents have greater nociceptive responses after hind paw injections of carrageenan^57^ and chronic constriction injury^58^. Whether a common mechanism contributes to female-preference pain diseases/modalities remains unknown.

In addition, OVX studies have presented inconsistent conclusions. Animals with OVX have been found to exhibit stronger abdominal hyperalgesia^59^, decreased pain thresholds in formalin-induced responses^60,61^, and glutamate-induced TMJ pain^62^, as compared with animals without OVX, whereas two earlier studies have reported different effects^63,64^. In contrast, ovary-intact rats present increased neuropathic pain^65^ and thermal hyperalgesia^56^ than do OVX rats. Another long-term survey has revealed that the OVX rats develop thermal hyperalgesia from 2 weeks to 7 weeks after OVX and mechanical allodynia at 5 weeks that persisted until 7 weeks^66^. Deprivation of oestrogen leading to different clinical characteristics has also been observed clinically^67^. These mutually contradictory studies have indicated that whether deprivation of ovarian hormones results in either pronociceptive or antinociceptive effects may depend on the types of stimuli and the duration of female hormone depletion.

The role of female hormones is unclear in females with chronic widespread muscle pain. Ovarian steroids may be a major factor determining sex-dependent nociception^33,34,68,69^, and oestrogen is involved in chronic pain syndromes^70^. Oestradiol also enhances capsaicin-induced acute pain^37^ and increases responsiveness to colorectal distension^71^. In contrast, antinociceptive actions of oestrogens have been postulated on the basis of different lines of evidence, including the observation, in long-term (8 weeks) ovariectomized mice, of persistent mechanical hyperalgesia that can be reversed by oestradiol replacement^72^, and the observation that oestradiol levels within physiological ranges attenuate CFA-induced TMJ pain^73^. In addition, during pregnancy, the maintenance of high levels of ovarian hormones decreases sensitivity to pain^74,75^. In the present study, we demonstrated that intrathecal injections of E2 in male rats and OVX rats produced transient mechanical allodynia (Figs 10, 11), thus demonstrating a pronociceptive effect. Again, these studies indicated that mechanism of female hormones in contributing to pronociception or antinociception is complicated and involving with multiple conditions.

Here, we provide the first report that ovarian hormone-dependent AIMP requires spinal activation of ERK. p-ERK activity was increased in the female rats early after AI and was maintained at high levels, in accordance with pain course and pain intensity. Inhibition by injection of an ERK inhibitor significantly decreased AIMP in normal female rats but had no effect on AIMP in OVX rats.

MAPK activation contributes to the initiation, development, and maintenance of nociceptive hypersensitivity in various pain models^22^. In particular, p-ERK is a hallmark of nociceptive sensitization in inflammatory and neuropathic pain^76,77^. ERK in spinal neurons is immediately activated in response to nociceptive firing, and p-ERK subsequently triggers intracellular cascades and downstream mediators, thereby resulting in central sensitization^77,78^. Of particular note, late ERK activation in spinal astrocytes has been suggested to maintain nociceptive sensitization and modulate long-term plasticity^79^. Zhuang *et al*.^80^ found that p-ERK was sequentially expressed in neurons (within hours), microglia (2 days), and astrocytes (21 days) in the superficial dorsal horn after spinal nerve ligation, and concluded that three types of cells (neuron, microglia, and astrocytes) can play distinct roles in the temporal evolution of neuropathic pain. We found similar phenomenon in this study though we did not purposely distinguish the cell-type differences.

As the precursors of p-ERK, ERK1/2 increased in the bilateral spinal cord of ovary-intact rats after acid injections. In the ipsilateral spinal dorsal horn, both p-ERK subtypes (p-ERK1 and p-ERK2) gradually increased to maximum levels on post-injection day 6, reaching a level significantly higher than pre-injection levels, and then decreased 14 days later. The changes in protein levels paralleled the increase in allodynic hypersensitivity at the injected paw, thus suggesting that ERK activation may control the development of AI-induced nociceptive behaviours. In addition, p-ERK activity may reflect expansion of sensitized receptive field, because ERK phosphorylation evenly appeared at the bilateral spinal dorsal horns for at least 2 weeks. Although western blot analysis indicated only unilateral increase in the injection-side, this finding was not surprising, because spinal p-ERK expression was clustered in small foci (i.e., lamina I and II) in proportion to the large dorsal horn mass, and differences in the contralateral side were highly diluted to an insignificant level. Such topographical identification further confirmed that AI actually induced widespread spinal sensitization.

Another intriguing finding was that p-ERK in the OVX rats was lower in comparison with that in the ovary-intact rats after acid injections, though ERK expressions in both group showed no statistical differences. In addition, the ERK inhibitor (U0126) attenuated AIMP levels in normal female rats but not in OVX rats. Further, AI-treated male rats expressed p-ERK1/2. Accordingly, p-ERK is required for female hormone-dependent AIMP in female rats and may also contribute to AIMP in male rats. Because intrathecal E2 or P4 in males induced mechanical allodynia, we thus propose that an ovarian hormone-dependent component of AIMP exists in rats, which is primarily mediated by p-ERK expression.

Min *et al*. have demonstrated that increased ERK activation in two supraspinal areas, the central amygdala and the paraventricular thalamic nucleus anterior, accounts for central sensitization in acid injection-induced CWP/FMS^81^. These authors have suggested that Cav3.2 T-type Ca^2+^ channel-dependent ERK activation in the paraventricular thalamus contributes to acid-induced CWP^31^. An increase of p-ERK in capsular central amygdaloid (CeAC) was observed in AIMP mice and involved in enhancing synaptic transmission of nociceptive parabrachio-amygdaloid (PBA) input onto CeAC^30^. Sluka’s group has also reported changes in phosphorylated CREB^20^ and concentrations of excitatory amino acids^21^ at the spinal cord level in acid-induced chronic hyperalgesia. Recently another evidence showing that ERK activation is involved in complicated spinal mechanisms related to microglia and astrocyte in sex-dependent pain responses^82^. Here, we provided new evidence by demonstrating the spinal role of p-ERK in female widespread pain and an involvement of spinal neurons and astrocytes in regulating chronic nociceptive hypersensitivity. Our results echo p-ERK functions in the brain and substantiate the importance of p-ERK in oestrogen-dependent AIMP.

Some experimental limitations might have created interpretation biases in this study. We did not thoroughly differentiate serum and CSF concentrations of E2 and P4 in OVX and normal rats, which may be critical in determining activity. The menstrual cycle may be another important factor influencing painful disorders in women. Fluctuating hormonal levels during the oestrous cycle affects pain sensitivity in rodents^83^. However, previous experiments has revealed that female mammals in proximity develop synchronized cycles^84,85^. Further, a technical limitation in this study may cause our immunostaining measurement not accurately reflect total p-ERK expression. We captured the images at a magnification of 20× objective to obtain photos at a resolution of 1936×1460 pixels and identified p-ERK-positive cells showing solid round, oval, or linear signals. This method, though carefully conducted, could still favour the inclusion of neuronal p-ERK but overlook astrocytic p-ERK because some fibrillary pERK signals in astrocytes may not be easily distinguished from the background staining. Such underestimate of astrocytic p-ERK may partially explain the different results between western blot and immunostaining.

In conclusion, this study provides the first demonstration of the involvement of ovarian hormones in an acid injection-induced muscle pain and addresses the important role of spinal ERK activation in the processes. We propose a novel perspective that ERK activation is required for ovarian hormone-dependent chronic widespread muscle pain or fibromyalgia in females. These findings may explain the sex differences in fibromyalgia and open new avenues for fibromyalgia treatment.

## Materials and Methods

### Animal preparation

Female and male Sprague-Dawley rats (2 months old) weighing approximately 200 g were purchased from BioLASCO (Taipei, Taiwan). Rats were housed in groups of three at 22 °C ± 1 °C with a 12-hr dark-light cycle, and food was available *ad libitum*. The study was performed under the approval of the Animal Care and Use Committee, China Medical University, Taichung, Taiwan and strictly followed the NIH Guidelines.

### Ovariectomy and sham surgery in rats

Female rats were subjected to bilateral ovariectomy (OVX) or bilateral sham surgery (Sham) under 1% isoflurane anaesthesia. Briefly, skin incisions approximately 1 cm in length were generated 2 cm from back midline to bilateral inferior borders of the ribs. The peritoneal cavity was opened, and the bilateral ovaries were removed. The skin and muscle incisions were closed with sterile nylon sutures. The female sham and the male sham (Male) operation rats received a similar surgery except no organs were removed. After surgery, rats were allowed to recover for at least 21 days. A previous study has reported that plasma oestrogen and progesterone concentrations were largely depleted 17 days after OVX surgery^86^, and concentrations were even lower than those in the oestrus stage^87^. To confirm the success of ovariectomy, serum 17β-oestradiol levels were determined before and after ovariectomy.

### Measurement of serum 17β-oestradiol (E2)

Blood samples were obtained by venipuncture of the tail under 1% isoflurane anaesthesia before bilateral ovariectomy, before the 1st acid injection (3 weeks post-surgery), and at day 14 after the 2nd acid injection (6 weeks post-surgery). The serum concentration of the unconjugated form of 17β-oestradiol was measured with a commercially available electrochemiluminescence immunoassay (ECLIA) kit “Elecsys Estradiol II” (Roche Diagnostics, Indianapolis, IN, USA). The detection limit is 5 pg/mL for ECLIA. Values below the detection limit were arbitrarily deemed 5 pg/mL for the statistical analysis.

### Repeated acid solution injection (AI)-induced widespread pain

The AI-induced CWP animal model was described previously^17^ and was slightly modified in this study. The HCl-saline solution at pH = 4 (ranged within 3.9 and 4.1) was prepared before the study. The acid HCl solution (150 μl) was injected into the left gastrocnemius muscle in rats on day 0 (D0) and day 5 (D5).

### Nociceptive behavioural tests

The mechanical threshold was evaluated by von Frey filaments (Stoelting, Wood Dale, IL). Animals were individually placed in a chamber (10 × 10 × 20 cm) of a Plexiglas cage on an elevated iron mesh floor. The von Frey filaments were applied from the mesh openings to stimulate the plantar surface by using the up-down method^88^. A series of von Frey filaments with logarithmically incremental stiffness (0.4, 1.0, 2.0, 4.0, 6.0, 10.0, 15.0, and 26.0 g) were presented perpendicularly to the plantar surface for 5–6 seconds for each filament. The threshold value is determined by calculation of the fifty percent withdrawal threshold^89^. Both hind paws were tested daily starting 2 days before the first acid injection, between two injections, and time points after the second injection. Baseline data were an average of two pre-injection measurements.

The thermal threshold was measured by paw withdrawal latencies to radiant heat stimulation. Rats were placed individually in the plantar test device (Plantar Test Apparatus, IITC, CA) with the glass floor pre-warmed to a constant 30 °C. After 15-min acclimation, a focused radiant heat source underneath the glass floor was projected to the paws. Cut-off latency was set at 30 s to avoid thermal injury. Each rat was tested three times at 5-min intervals, and the measurements were averaged to represent the withdrawal latency. Data were obtained at the same time points for the von Frey test.

### Intrathecal injection of the ERK MAPK inhibitor in female rats

To investigate the involvement of ERK activation in nociceptive sensitization, i.t. injection of the ERK inhibitor U0126 (Sigma-Aldrich, Saint Louis, MO) was performed in female rats. On the 5th day after the 2nd AI (PD5), a direct dural puncture was performed at L4-5 or L5-6 interspace with a 30 G needle attached to a 25-μL Hamilton microsyringe under anaesthesia with 1% isoflurane. Correct needle placement inside the spinal thecal canal was confirmed by the appearance of a brief rapid tail flick. U0126 (10 μg in 20 μl 5% dimethyl sulphoxide, DMSO) or vehicle solution (20 μl, 5% DMSO) was then slowly injected. This i.t. dose has previously been shown to effectively inhibit complete Freund’s adjuvant-induced arthritic pain in rats^90^. Behavioural tests and spinal immunoassays were conducted to evaluate responses to the injection.

### Intrathecal injection of ovarian hormones in male rats

To investigate the effects of ovarian hormones on nociceptive responses of male rats, water-soluble E2 (Sigma-Aldrich, Saint Louis, MO, 100 nM in 10 μl normal saline), P4 (Sigma-Aldrich, Saint Louis, MO, 30 μM in 10 μl normal saline), or vehicle solution (10 μl, saline) was slowly i.t. injected into the L4-5 or L5-6 interspace in male rats. These E2^91^ and P4^92^ i.t. doses have been shown to facilitate the repetitive stimulation-induced spinal reflex effectively. Behavioural tests were conducted to evaluate responses to the injection.

### Intrathecal injection of ovarian hormones in OVX female rats

To investigate the effects of ovarian hormones on nociceptive responses of female rats, water-soluble E2 (Sigma-Aldrich, Saint Louis, MO, 100 nM in 10 μl normal saline) or vehicle solution (10 μl, saline) was twice injected into the L4-5 or L5-6 interspace in OVX female rats. Each E2 delivery was conducted one day before acid injection. Behavioural tests were conducted to evaluate responses to the injection.

### Western blotting

Animals were anaesthetized with a high concentration of isoflurane and sacrificed. The bilateral dorsal spinal cord at L4-5 segments was dissected. Tissue lysates were prepared by homogenization and fractionation into cytosolic, membrane, and nuclear fractions using a cytoplasmic, nuclear, and membrane compartment protein extraction kit, as recommended by the manufacturer (Biochain Institute, Inc., Hayward, Calif). Each cytosolic protein sample (20 μg) was separated on a 8 or 10% polyacrylamide-SDS gel in a glycine-Tris buffer, transferred to a PVDF membrane, and probed with rabbit anti-ERK and anti-p-ERK antibodies (1:1,000, Cell Signaling Technology, Danvers, MA) at 4 °C overnight. The immune complexes were further probed with a HRP-conjugated anti-rabbit IgG secondary antibody (1:5000, Jackson ImmunoResearch, West Grove, PA) and then visualized with HRP-reactive chemiluminescence reagents (Millipore Corporation, Billerica, Massachusetts, USA). The chemiluminescent band was detected and analysed with an ImageQuant LAS 4000 imaging system (GE Healthcare Bio-Sciences AB, Uppsala, Sweden). The band intensity of each group was normalized to that of GAPDH, and values are presented as the fold-change compared with control for statistical analysis. All the western blots were performed under a standardized procedure.

### Immunofluorescence staining

Rats were deeply anaesthetized and perfused intracardially with 4% paraformaldehyde in phosphate-buffered saline. The lumbar spinal cord was removed and cut into 30-μm transverse sections in a cryostat (LEICA CM3050S, Nussloch, Germany). After being washed with Tris-buffered saline (TBS), sections were blocked with 3% normal goat serum in TBS for 1 h and then incubated with rabbit anti-p-ERK antibody (1:400; Cell Signaling Technology, Danvers, MA) at 4 °C overnight. Sections were then reacted with Cy3-conjugated secondary antibody (1:400, Jackson ImmunoResearch, West Grove, PA) for 1 h at room temperature. For double staining, sections were incubated with a mixture of anti-p-ERK (1:400) and anti-NeuN (neuronal marker, 1:1500; Chemicon, Temecula, CA), anti-OX-42 (microglia marker, 1:200; Serotec, Indianapolis, IN), or anti-GFAP (astrocyte marker, 1:6000; Chemicon) antibodies from different species and then reacted with a mixture of Alexa 488- and Cy3-conjugated secondary antibodies (1:400, Jackson ImmunoResearch, West Grove, PA). Slides were air-dried and dehydrated, and cover slips were applied. All section images were captured using a CCD camera (ZEISS Axiocam 105 colour, Jena, Germany) connected to a Zeiss Axio Imager A2 microscope (Göttingen, German) or spectral confocal microscopy (Leica SP2, MAJOR, Germany), and the fluorescence intensity of the spinal section was analysed using ImageJ (1.47 v, NIH Image, National Institutes of Health, Bethesda, MD). All images were captured under a 20× objective in a square box (450 × 338 μm) using a standardized microscopic setting to obtain an image at a resolution of 1936 × 1460 pixels, and the top line of the square was parallel with the edge of the dorsal horn grey matter. The size of the box includes the medial two-thirds of the dorsal horn (laminae I–V) to represent the interested region where most p-ERK–immunoreactive (ir) cells were expressed after acid-injections. We calculated p-ERK-ir cells by Rexed laminae (I-V) on randomly chosen sections, using at least 8 for each spinal segment, and values were averaged for analysis. To have consistent results, all images were followed a standardized brightness adjustment and were universally converted to 8-bit greyscale. We defined positive cells which have solid round, oval, or linear morphology and distinct signals from background noise to show bright signal/noise ratio >2. For S/N ratio, we compared the intensity of a manually circled bright signals, the region of interest (ROI), with that of a dark spot with the same size and shape at adjacent background. Colocalization analysis used images taken by confocal microscope to have better resolution. The observer was unaware of the treatments during counting.

### Study deign

#### Experiment 1: Effect of ovariectomy on AIMP in female rats

The sham group and the ovariectomy (OVX) group were both subjected to repeated AI 3 weeks after surgery. Behavioural tests were conducted before AI as baseline (BL) data; immediately before the first AI (D0) and second AI (D5); and at 3 hours, day 1, day 3, day 6, day 8, and day 14 after the second AI.

#### Experiment 2: Role of ERK activation in AIMP

The Sham and OVX rats were sacrificed on days 1, 6, and 14 after the 2nd AI for immunostaining and western blot analysis. In addition, the Sham or OVX groups received i.t. injections of 10 μg of U0126 or 20 μL of 10 μg vehicle on Day 5 after 2nd AI (PD5) to assess behavioural and p-ERK responses.

#### Experiment 3: Sex effects on AIMP and ERK activation

Behavioural and p-ERK responses to repeated AIs were compared among the Sham, OVX, and sham male rats. In a separate study, E2 was i.t. injected to OVX rats to detect the hormonal effect in the AIMP model. Simultaneously, E2 and P4 were i.t. injected in male rats to evaluate the effects of ovarian hormones on pain responses.

### Statistical analysis

Data are presented as the mean ± standard error of the mean (SEM). Behavioural observations were analysed by two-way repeated measures- (RM-) ANOVA followed by *post hoc Tukey*’*s* test for group differences and one-way RM ANOVA for time course change. Relative protein expression levels and immunoreactive cell counts at different time points were analysed by one-way ANOVA and followed by *post hoc Tukey*’*s* test or unpaired Student’s *t-*test when appropriate for multiple groups. Statistical software GraphPad Prism v.2 (GraphPad Software, Inc., San Diego, CA) was used for calculation. Differences were considered significant when the p-value was less than 0.05.

## Supplementary information

Supplementary
