# Supplementary material for: Ovarian Hormone-dependent and Spinal ERK Activation-regulated Nociceptive Hypersensitivity in Female Rats with Acid Injection-induced Chronic Widespread Muscle Pain
Source: Sci Rep. 2019 Feb 28;9:3077. doi: 10.1038/s41598-019-39472-z (PMC6395742; doi:10.1038/s41598-019-39472-z)
Supplement: Supplementary file 1 — Supplementary [file 41598_2019_39472_MOESM1_ESM.pdf]

Ovarian Hormone-dependent and Spinal ERK Activation-regulated Nociceptive Hypersensitivity in Female Rats with Acid Injection-induced Chronic Widespread Muscle Pain

Ju-Hsin Chang[1,2], Shih-Ying Tsai[3], Yen-Jing Zeng[1], Yu-Cheng Liu[2], Chi-Yuan Li[1,2], Kuen-Bao Chen[2], Yeong-Ray Wen[2,3,4,5]

[1]Graduate Institute of Clinical Medical Science, China Medical University, Taichung, Taiwan.
[2]Department of Anesthesiology, China Medical University Hospital, Taichung, Taiwan.
[3]Department of Anesthesiology, School of Medicine, China Medical University, Taichung, Taiwan.
[4]Graduate Institute of Acupuncture Science, College of Chinese Medicine, China Medical University, Taichung, Taiwan.
[5]Acupuncture Research Center, China Medical University, Taichung, Taiwan

Corresponding Author:
Yeong-Ray Wen, M.D., Ph.D.

Supplemental Figure S1-1

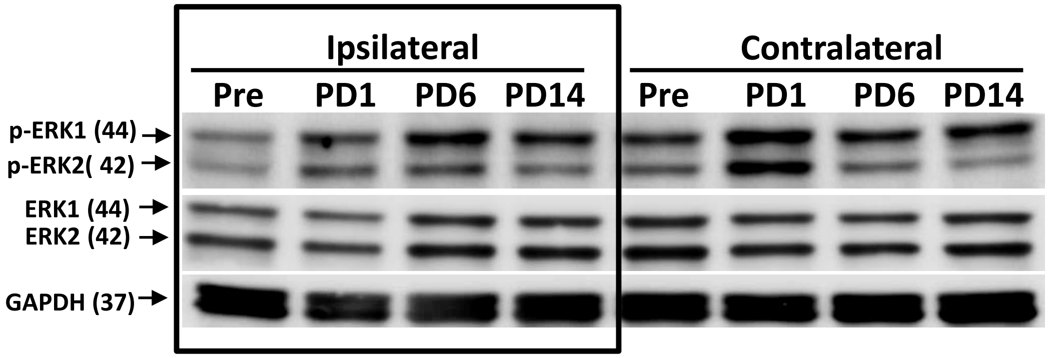

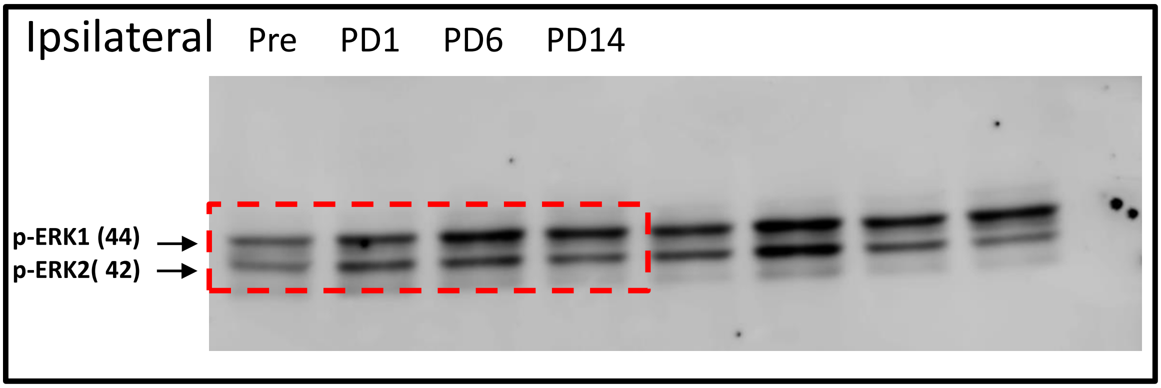

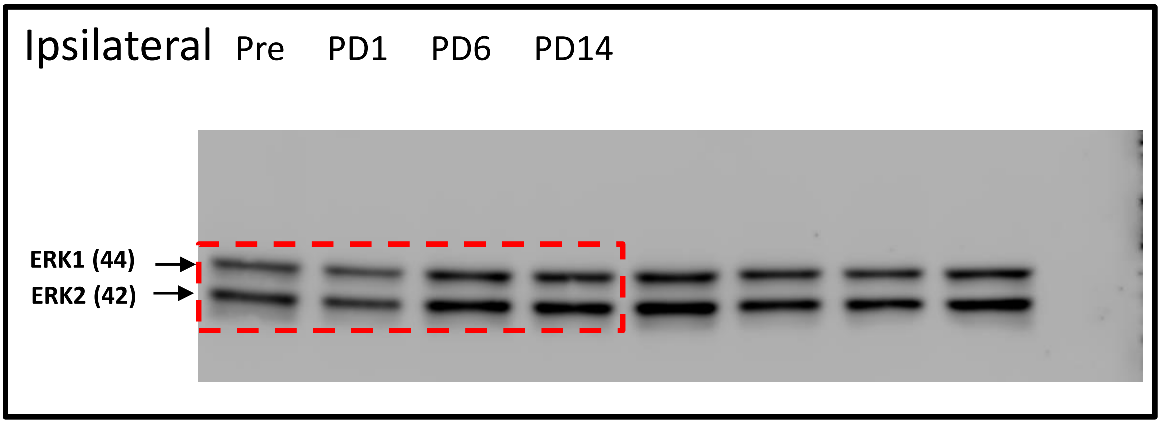

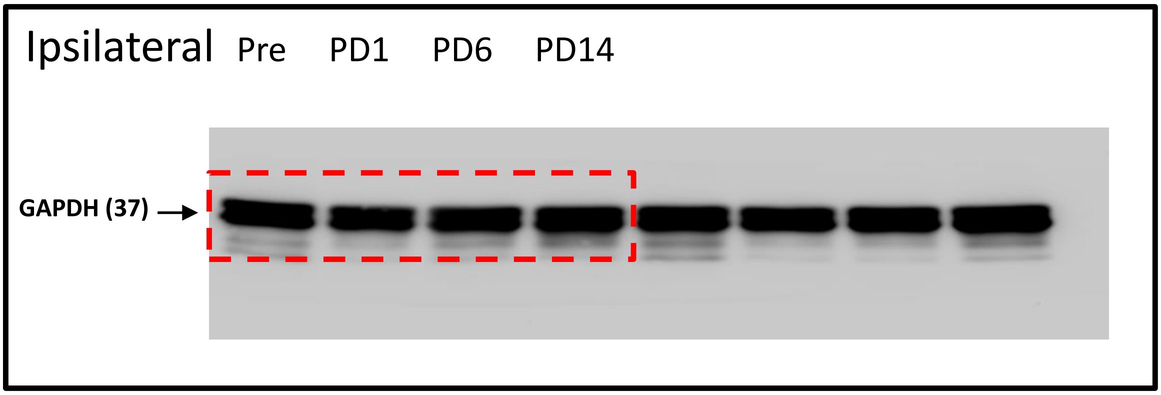

Supplemental Figure S1-1. Full length blots of figure 3 ipsilateral panel. Red dotted lines show the cropping locations.

Supplemental Figure S1-2

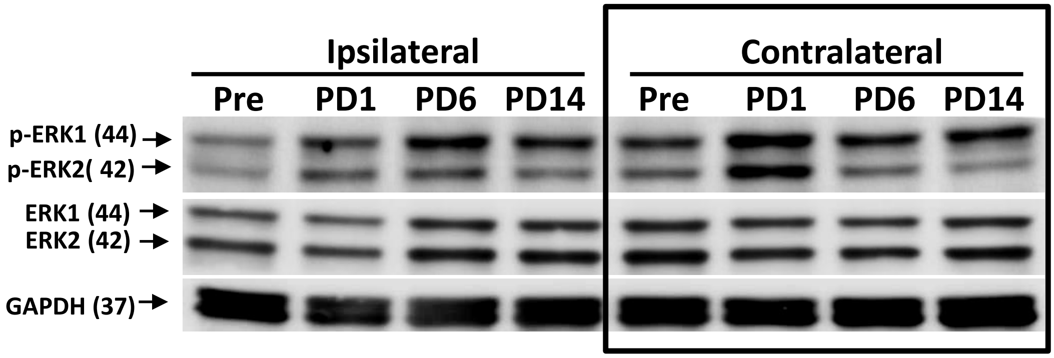

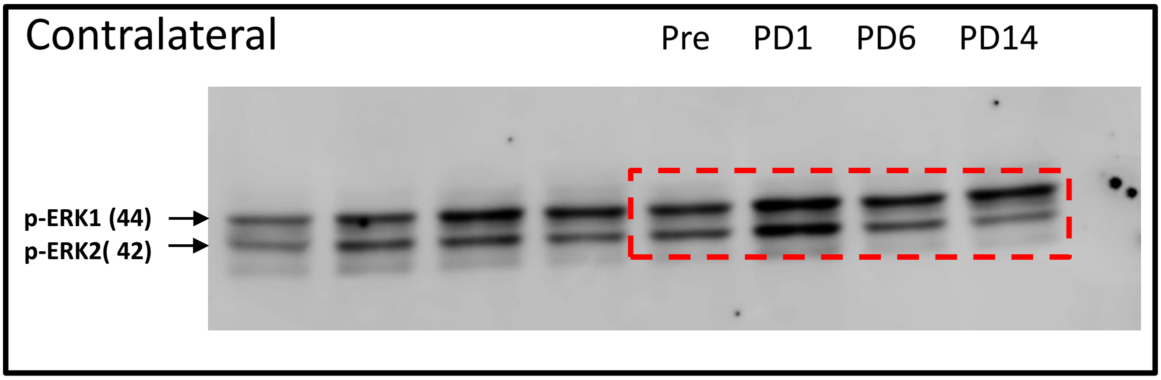

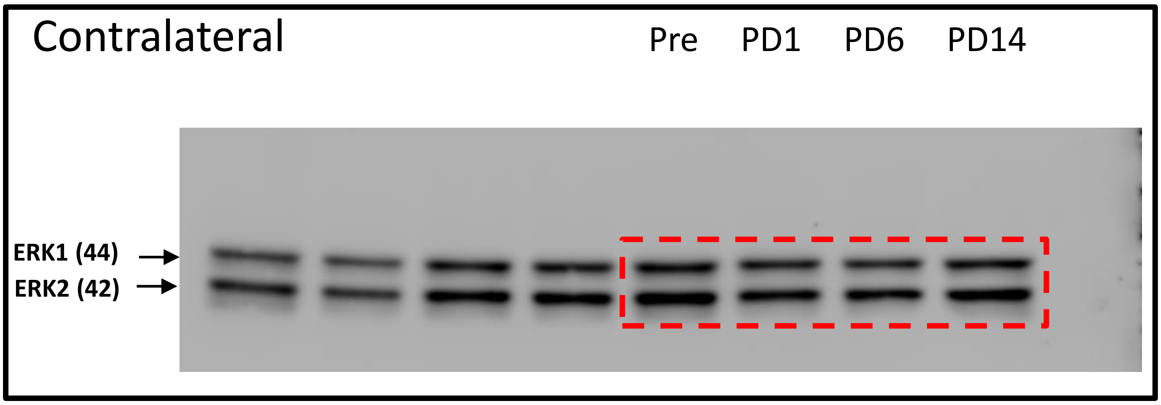

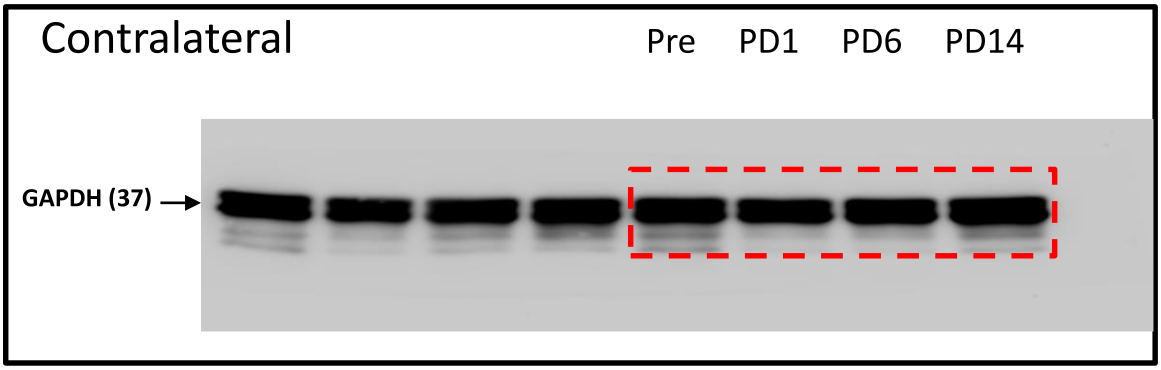

Supplemental Figure S1-2. Full length blots of figure 3 contralateral panel. Red dotted lines show the cropping locations.

Supplemental Figure S2-1

A. 1 day post 2nd acid

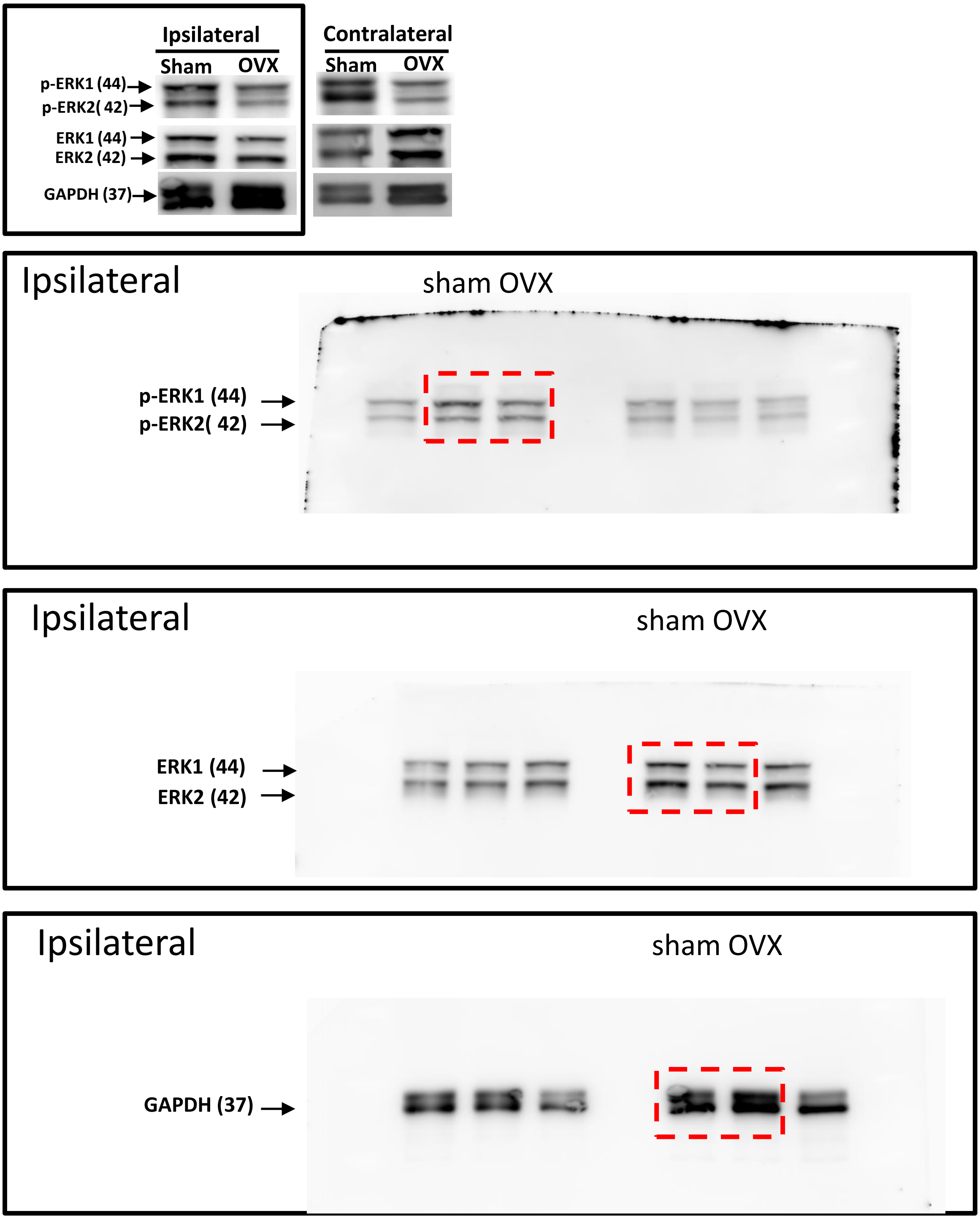

Supplemental Figure S2-1. Full length blots of figure 4A ipsilateral panel. Red dotted lines show the cropping locations.

Supplemental Figure S2-2

A. 1 day post 2nd acid

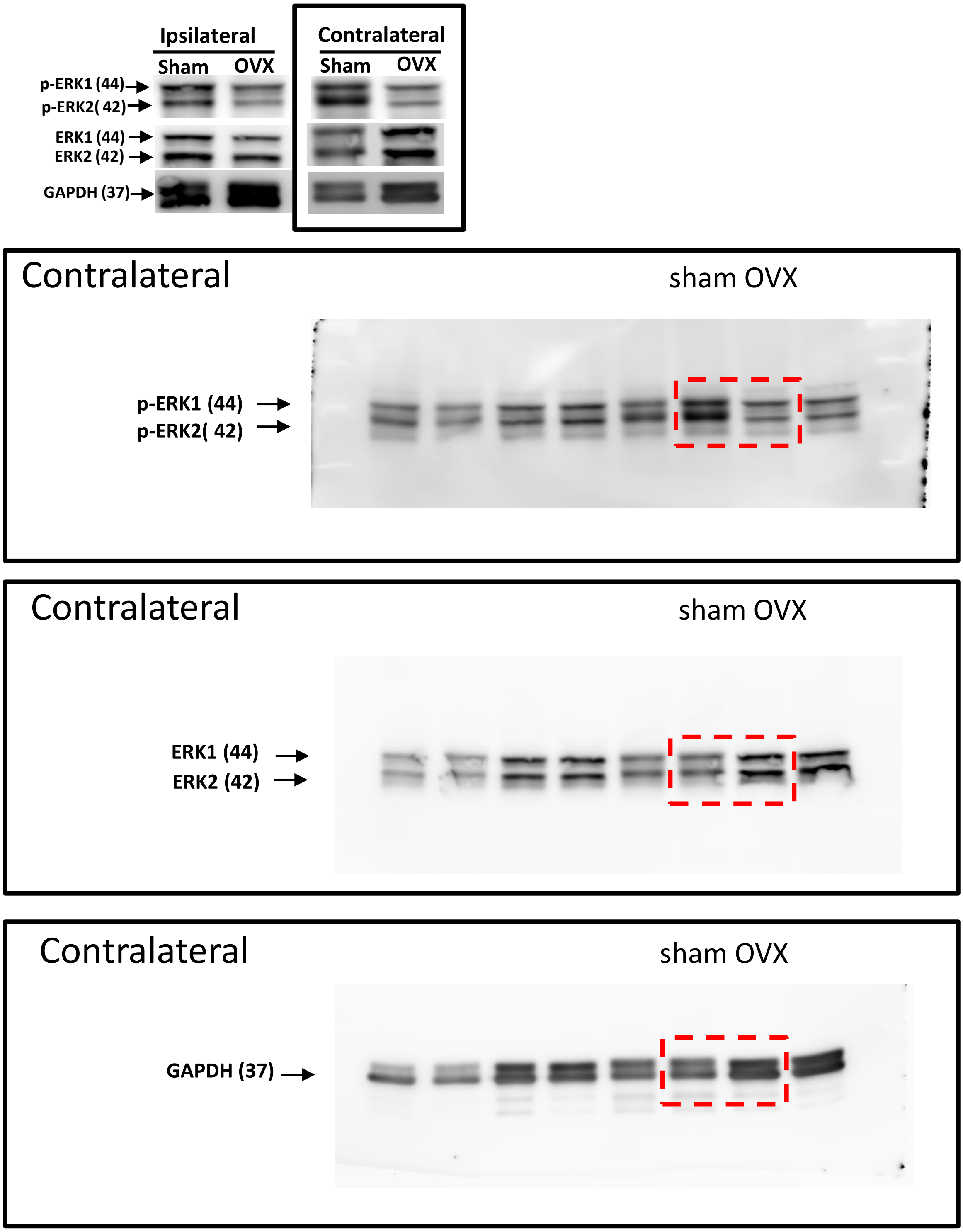

Supplemental Figure S2-2. Full length blots of figure 4A contralateral panel. Red dotted lines show the cropping locations.

Supplemental Figure S2-3

B. 6 day post 2nd acid

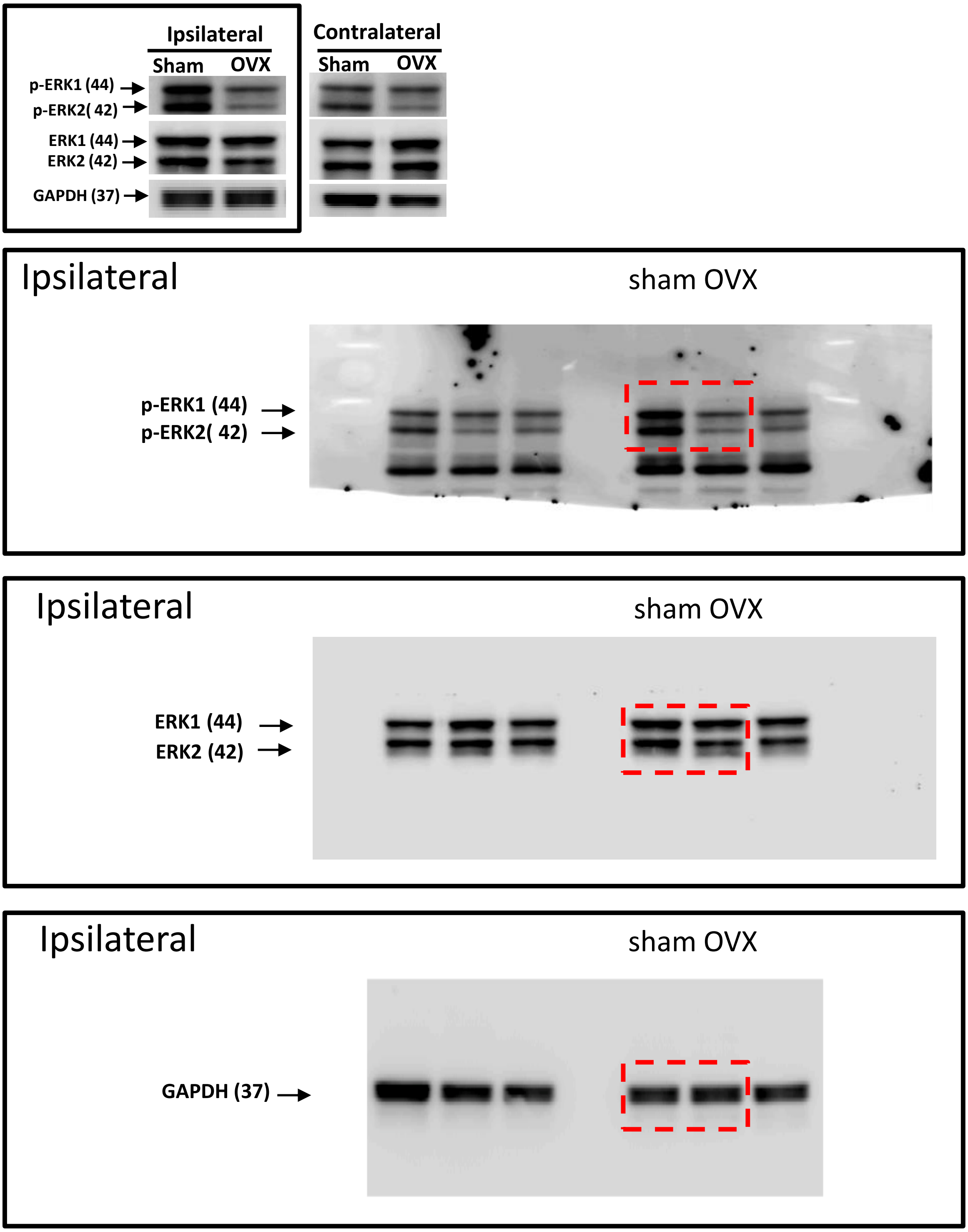

Supplemental Figure S2-3. Full length blots of figure 4B ipsilateral panel. Red dotted lines show the cropping locations.

Supplemental Figure S2-4

B. 6 day post 2nd acid

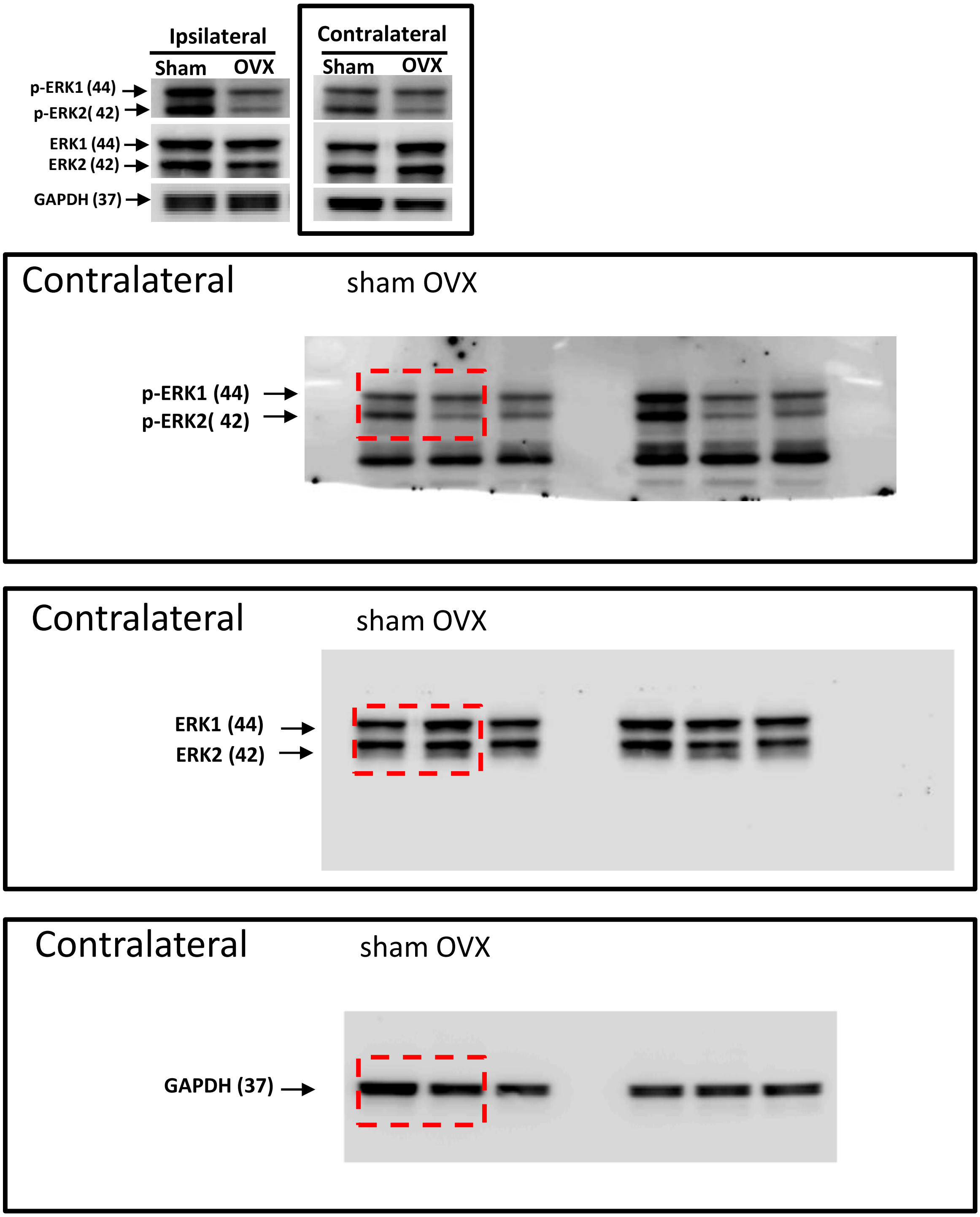

Supplemental Figure S2-4. Full length blots of figure 4A contralateral panel. Red dotted lines show the cropping locations.

Supplemental Figure S2-5

C. 14 day post 2nd acid

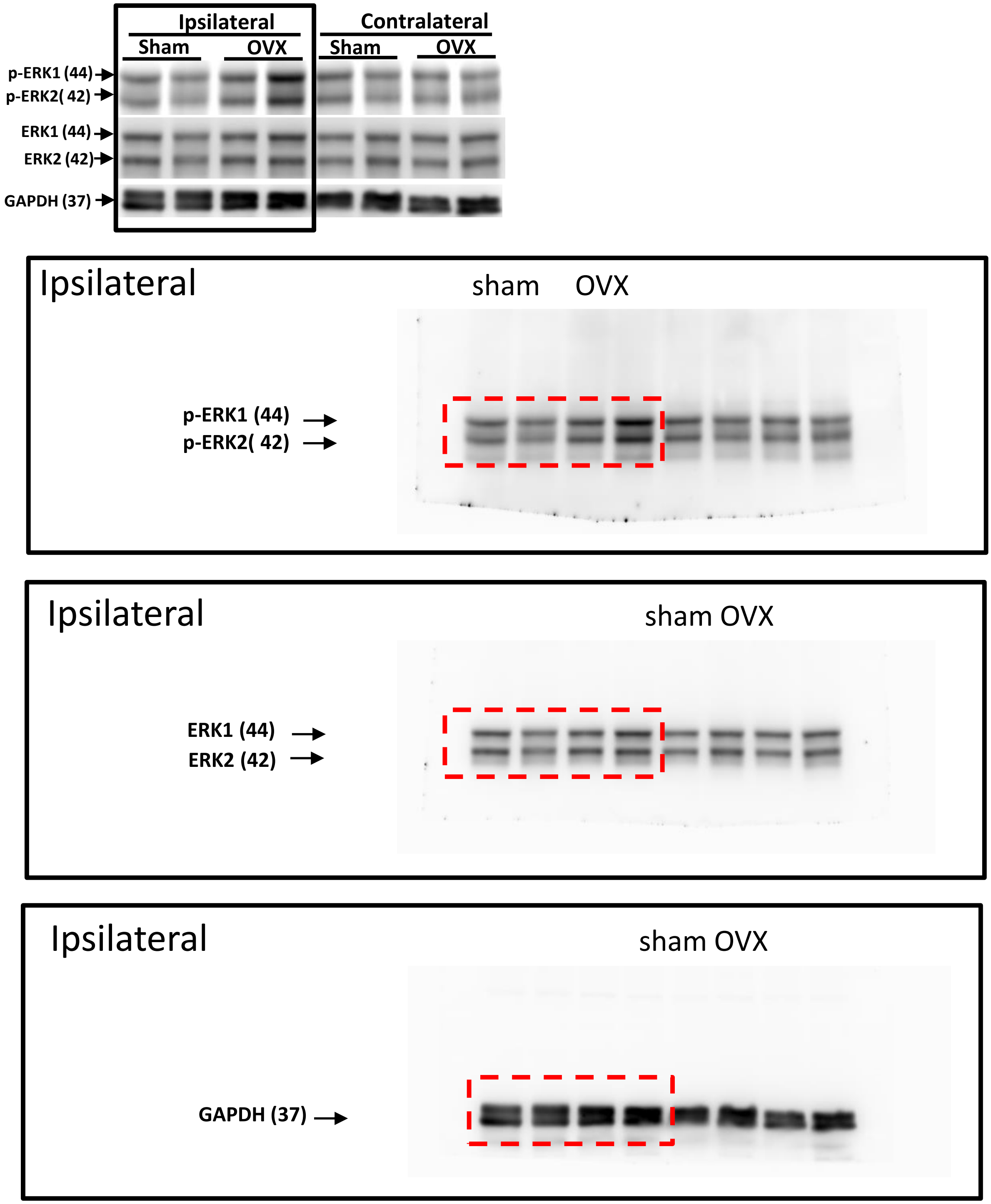

Supplemental Figure S2-5. Full length blots of figure 4C ipsilateral panel. Red dotted lines show the cropping locations.

Supplemental Figure S2-6

C. 14 day post 2nd acid

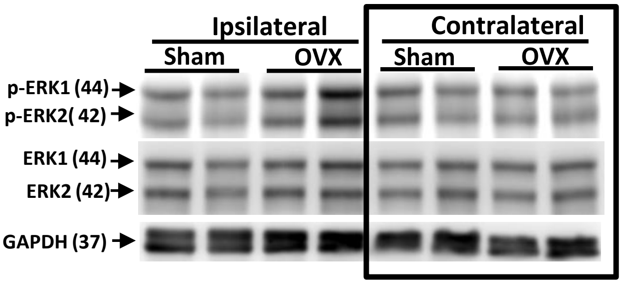

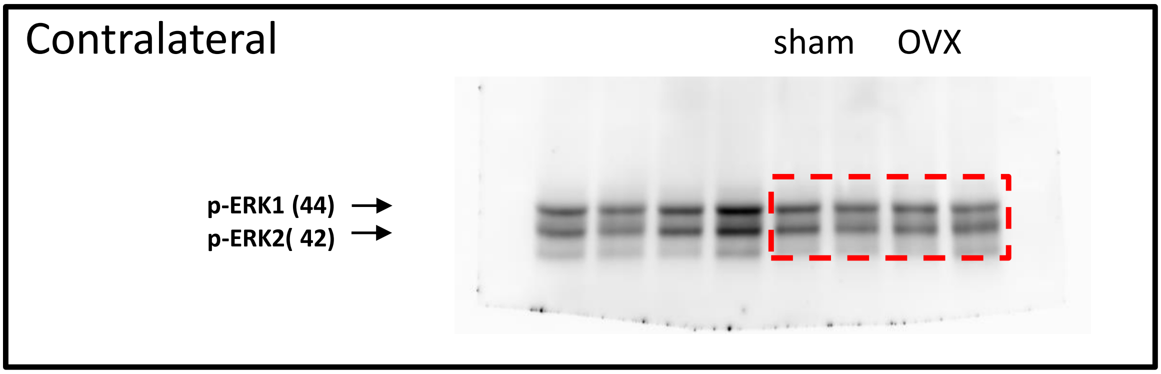

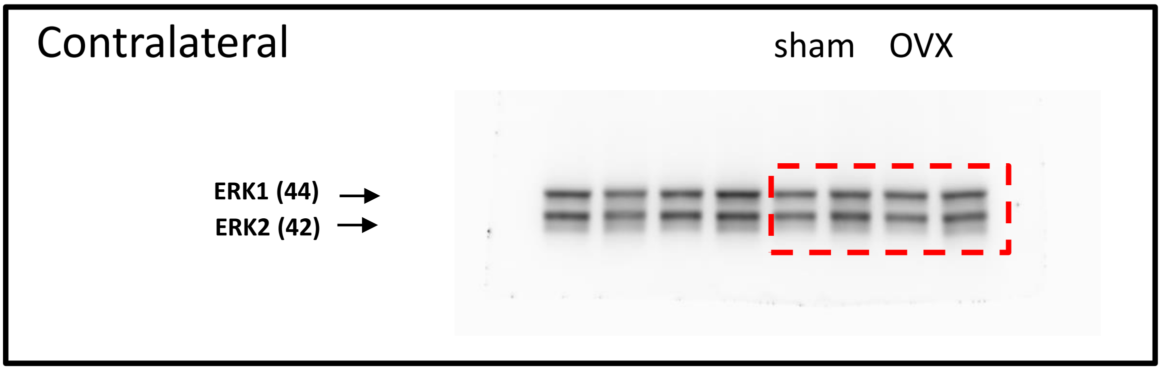

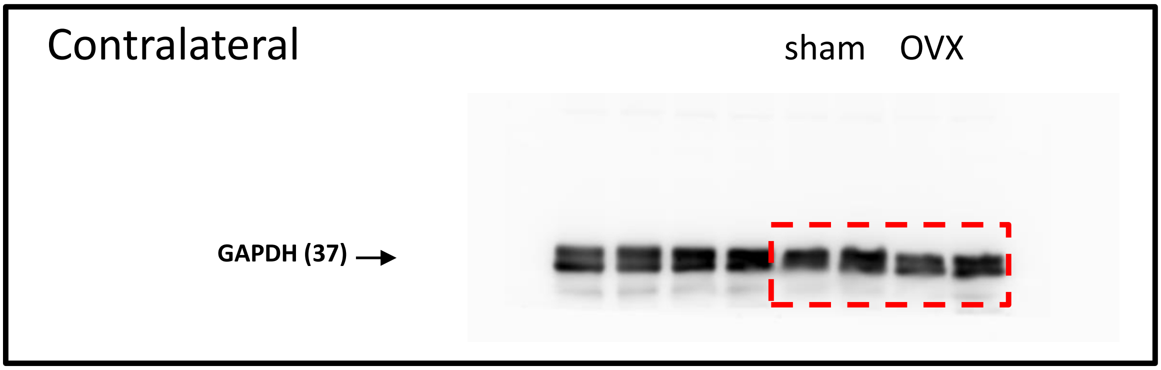

Supplemental Figure S2-6. Full length blots of figure 4C contralateral panel. Red dotted lines show the cropping locations.

Supplemental Figure S3

A. 1 day post 2nd acid

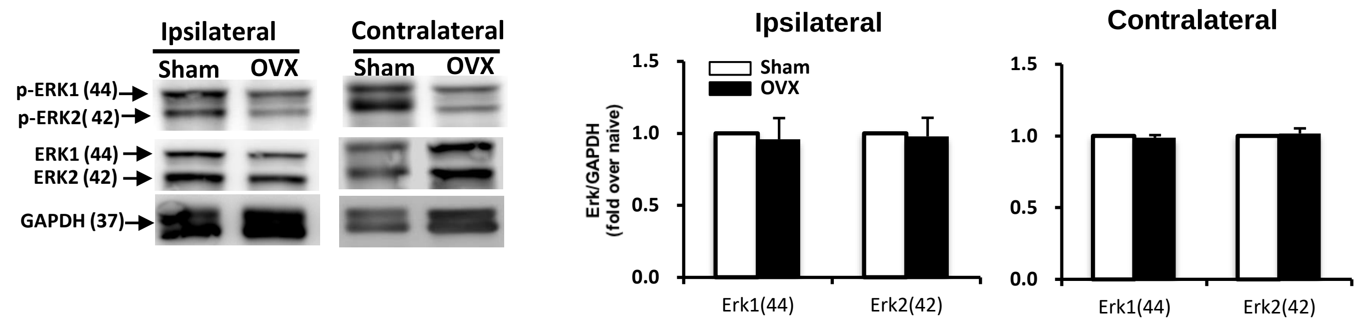

B. 6 day post 2nd acid

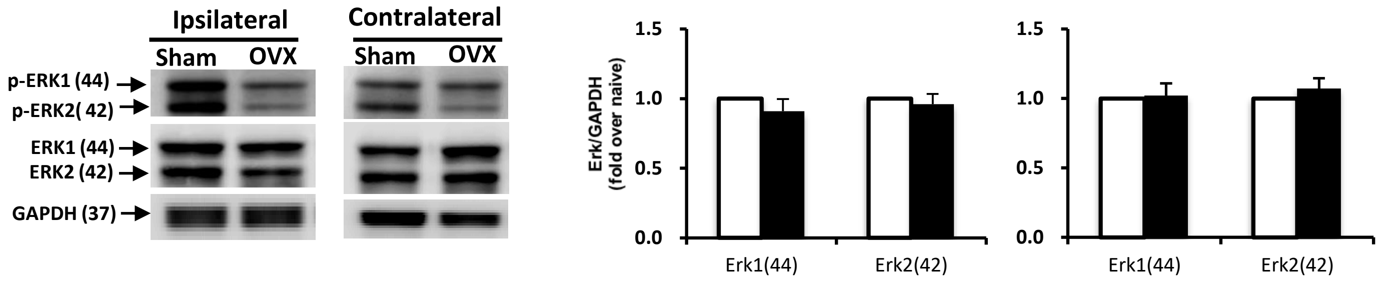

C. 14 day post 2nd acid

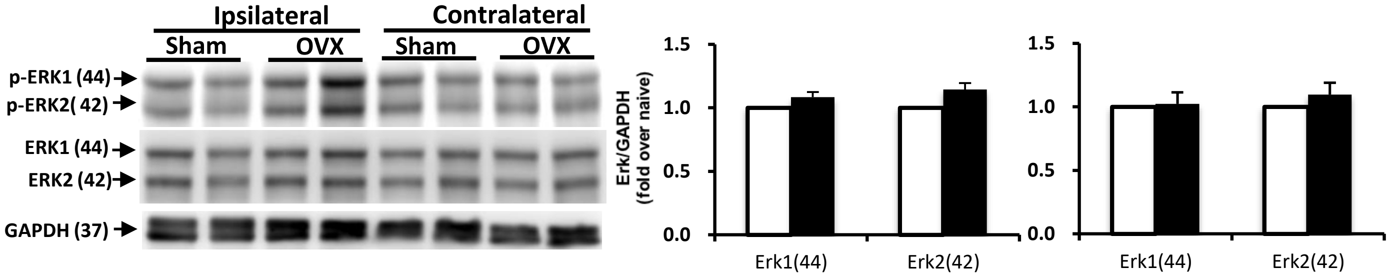

Supplemental Figure S3. Influence of ovariectomy on ERK expression. Western blots (left) and quantification (right) of p-ERK1/2 and ERK1/2 in the dorsal cords of the sham and OVX rats on days 1, 6, and 14 after the 2nd AI are presented. In the right panels, the densities of ERK1/2 bands are normalized to GAPDH and presented as fold-change compared with sham rats. * $p < 0.05$; ** $p < 0.01$ vs. sham by unpaired *t* test. Rat number = 4-6 for each group at individual time points.

B. 6 day post 2nd acid **Supplemental Figure S4-1**

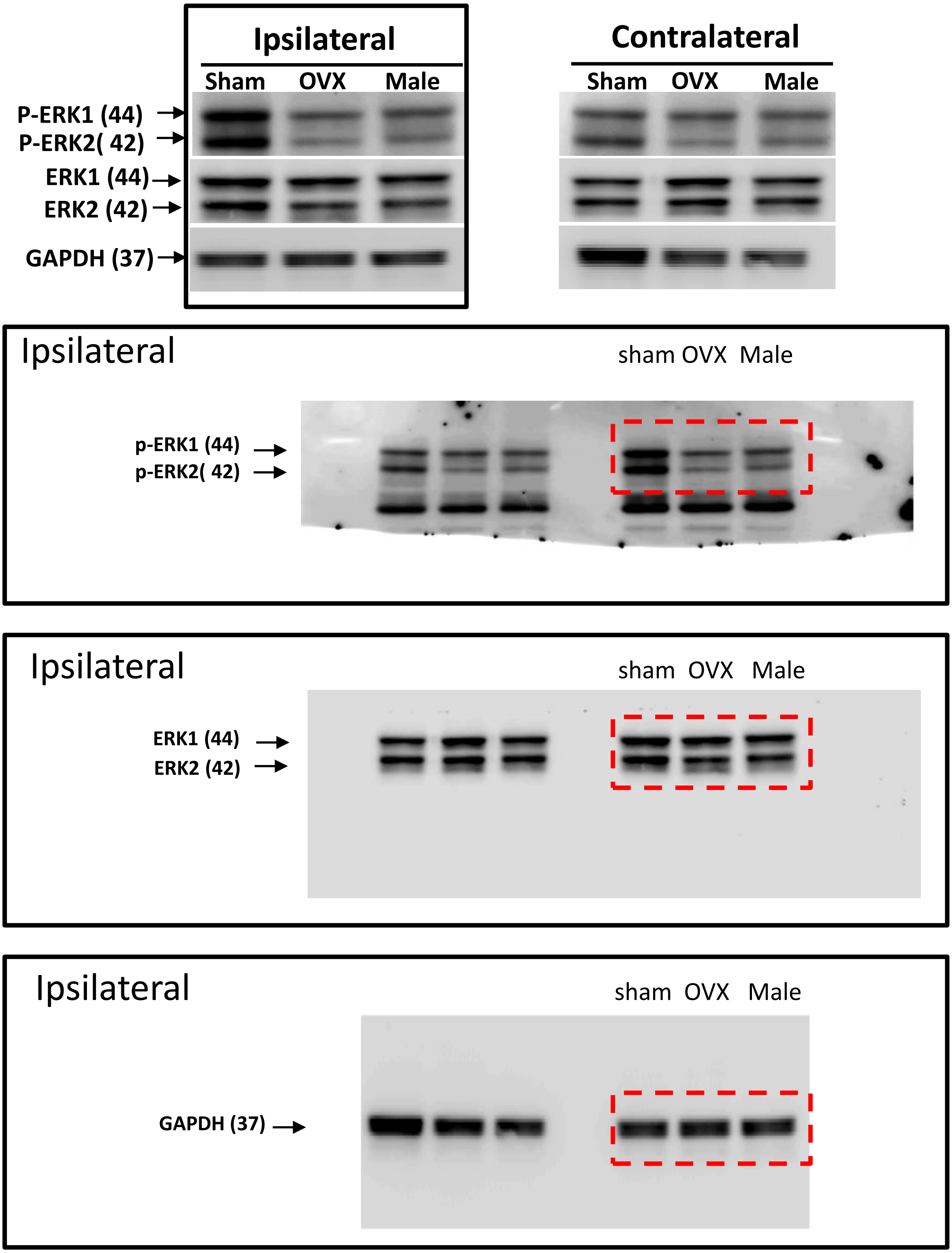

Supplemental Figure S4-1. Full length blots of figure 9 ipsilateral panel. Red dotted lines show the cropping locations.

B. 6 day post 2nd acid

Supplemental Figure S4-2

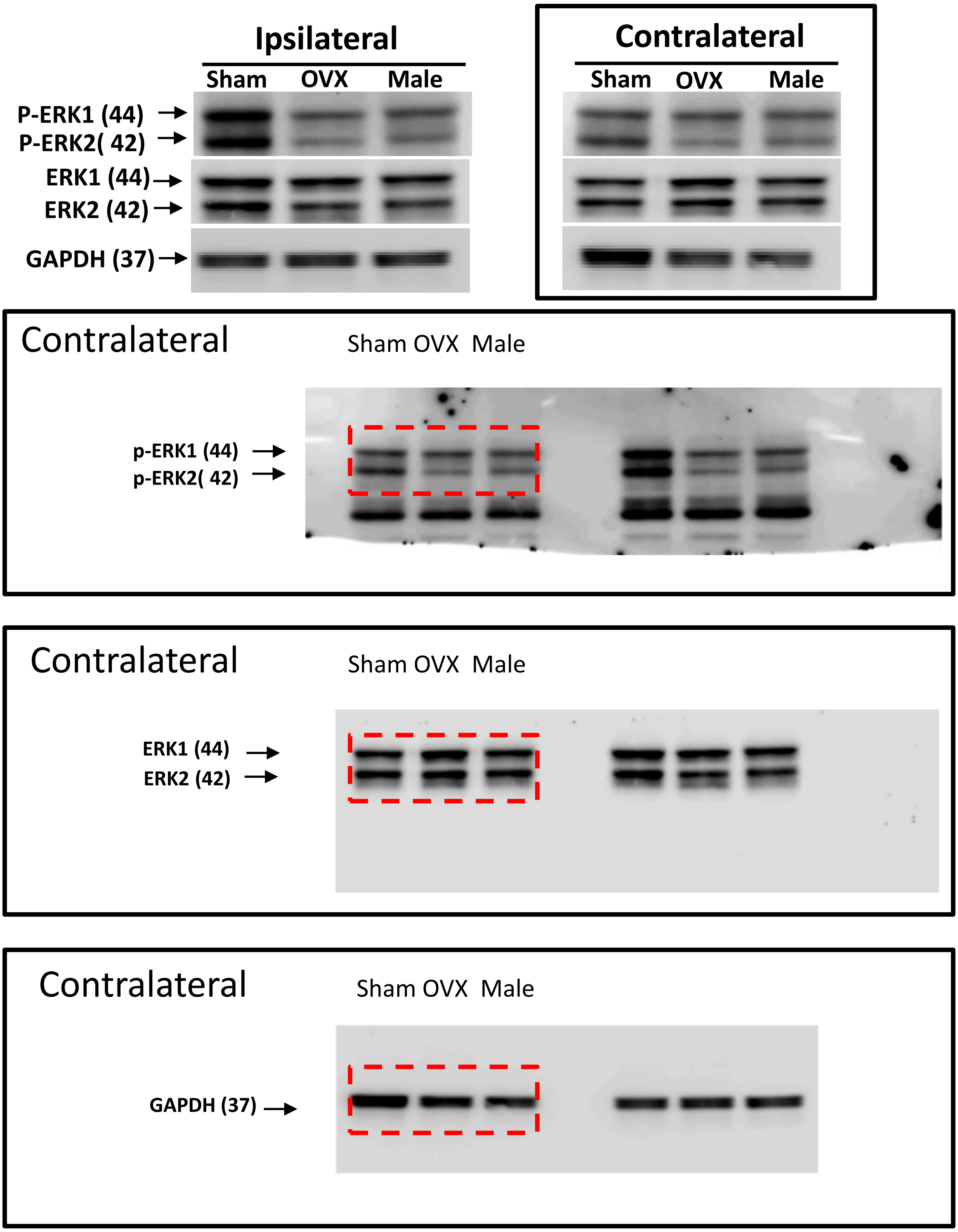

Supplemental Figure S4-2. Full length blots of figure 9 contralateral panel. Red dotted lines show the cropping locations.

Supplemental Figure S5

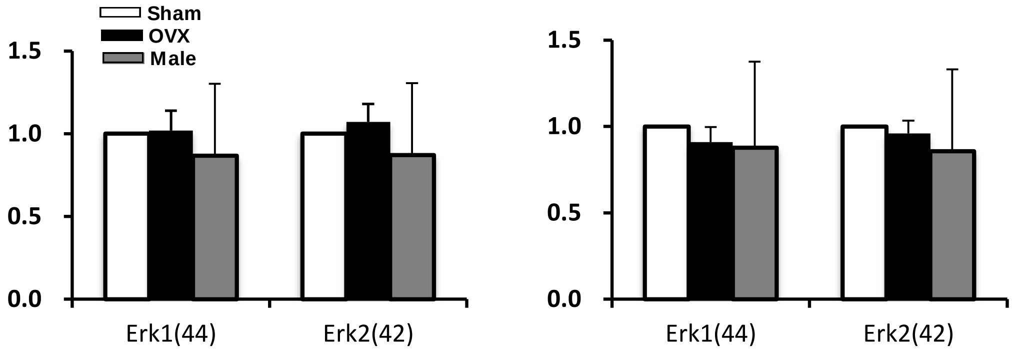

Supplemental Figure S5. Male rats displayed similar ERK1/2 expression after AIs than did female rats.
